# Antivirals for treatment of severe influenza: a systematic review and network meta-analysis of randomised controlled trials

**DOI:** 10.1016/S0140-6736(24)01307-2

**Published:** 2024-08-24

**Authors:** Ya Gao, Gordon Guyatt, Timothy M Uyeki, Ming Liu, Yamin Chen, Yunli Zhao, Yanjiao Shen, Jianguo Xu, Qingyong Zheng, Zhifan Li, Wanyu Zhao, Shuyue Luo, Xiaoyan Chen, Jinhui Tian, Qiukui Hao

**Affiliations:** a Evidence-Based Medicine Center, School of Basic Medical Sciences, Lanzhou University, Lanzhou, China; b Department of Health Research Methods, Evidence, and Impact, McMaster University, Hamilton, ON, Canada; c Department of Medicine, McMaster University, Hamilton, ON, Canada; d School of Rehabilitation Science, McMaster University, Hamilton, ON, Canada; e MAGIC Evidence Ecosystem Foundation, Oslo, Norway; f Influenza Division, US Centers for Disease Control and Prevention, Atlanta, GA, USA; g Clinical Nursing Teaching and Research Section, The Second Xiangya Hospital, Central South University, Changsha, China; h Xiangya School of Nursing, Central South University, Changsha, China; i Department of Geriatric Medicine, The Second Affiliated Hospital of Chongqing Medical University, Chongqing, China; j Chongqing Municipality Clinical Research Center for Geriatrics, The Second Affiliated Hospital of Chongqing Medical University, Chongqing, China; k Chinese Evidence-Based Medicine Center, West China Hospital, Sichuan University, Chengdu, China; l The First Clinical Medical College of Lanzhou University, Lanzhou, China; m National Clinical Research Centre for Geriatrics, West China Hospital, Sichuan University, Chengdu, China; n Center of Gerontology and Geriatrics, West China Hospital, Sichuan University, Chengdu, China; o Department of Geriatric, Zigong Affiliated Hospital of Southwest Medical University, Zigong, China

## Abstract

**Background:**

The optimal antiviral drug for treatment of severe influenza remains unclear. To support updated WHO influenza clinical guidelines, this systematic review and network meta-analysis evaluated antivirals for treatment of patients with severe influenza.

**Methods:**

We systematically searched MEDLINE, Embase, Cochrane Central Register of Controlled Trials, Cumulative Index to Nursing and Allied Health Literature, Global Health, Epistemonikos, and ClinicalTrials.gov for randomised controlled trials published up to Sept 20, 2023, that enrolled hospitalised patients with suspected or laboratory-confirmed influenza and compared direct-acting influenza antivirals against placebo, standard care, or another antiviral. Pairs of coauthors independently extracted data on study characteristics, patient characteristics, antiviral characteristics, and outcomes, with discrepancies resolved by discussion or by a third coauthor. Key outcomes of interest were time to alleviation of symptoms, duration of hospitalisation, admission to intensive care unit, progression to invasive mechanical ventilation, duration of mechanical ventilation, mortality, hospital discharge destination, emergence of antiviral resistance, adverse events, adverse events related to treatments, and serious adverse events. We conducted frequentist network meta-analyses to summarise the evidence and evaluated the certainty of evidence using the GRADE (Grading of Recommendations Assessment, Development and Evaluation) approach. This study is registered with PROSPERO, CRD42023456650.

**Findings:**

Of 11 878 records identified by our search, eight trials with 1424 participants (mean age 36–60 years for trials that reported mean or median age; 43–78% male patients) were included in this systematic review, of which six were included in the network meta-analysis. The effects of oseltamivir, peramivir, or zanamivir on mortality compared with placebo or standard care without placebo for seasonal and zoonotic influenza were of very low certainty. Compared with placebo or standard care, we found low certainty evidence that duration of hospitalisation for seasonal influenza was reduced with oseltamivir (mean difference –1·63 days, 95% CI –2·81 to –0·45) and peramivir (–1·73 days, –3·33 to –0·13). Compared with standard care, there was little or no difference in time to alleviation of symptoms with oseltamivir (0·34 days, –0·86 to 1·54; low certainty evidence) or peramivir (–0·05 days, –0·69 to 0·59; low certainty evidence). There were no differences in adverse events or serious adverse events with oseltamivir, peramivir, and zanamivir (very low certainty evidence). Uncertainty remains about the effects of antivirals on other outcomes for patients with severe influenza. Due to the small number of eligible trials, we could not test for publication bias.

**Interpretation:**

In hospitalised patients with severe influenza, oseltamivir and peramivir might reduce duration of hospitalisation compared with standard care or placebo, although the certainty of evidence is low. The effects of all antivirals on mortality and other important patient outcomes are very uncertain due to scarce data from randomised controlled trials.

**Funding:**

World Health Organization.

## Introduction

Influenza, a viral respiratory disease, typically causes mild to moderate upper respiratory symptoms that resolve within a week.^1^, ^2^, ^3^ However, a substantial proportion of individuals, particularly those in groups at high risk, such as young children (<5 years), older adults (≥65 years), pregnant women, and people with chronic medical conditions, can develop severe illness from influenza.^1^, ^4^

Influenza is an important cause of respiratory viral disease among hospitalised patients, resulting in hundreds of thousands of respiratory deaths worldwide annually, and major economic losses.^5^, ^6^, ^7^, ^8^ Hospitalised patients with seasonal influenza can develop complications, including severe pneumonia, respiratory failure, multi-organ failure, and secondary bacterial infections, that can lead to death.^1^, ^9^, ^10^, ^11^, ^12^ The case-fatality proportion for adults hospitalised with influenza typically ranges from 4% to 8%, but might be higher (10–15% or higher) during rare pandemics and among immunocompromised individuals.^13^ Therefore, identifying effective therapies for severe influenza is of global public health importance.

**Research in context**

**Evidence before this study**

Antivirals are frequently used in the clinical management of people with severe influenza. Previous systematic reviews and meta-analyses have reported that early initiation of neuraminidase inhibitor treatment in hospitalised patients with influenza might be associated with reduced mortality and length of hospital stay compared with later or no neuraminidase inhibitor treatment. However, these pairwise meta-analyses mainly focused on the relative effects of one specific class of antivirals (neuraminidase inhibitors), did not evaluate the effects of antivirals on severe zoonotic influenza, and did not assess the certainty of evidence. To our knowledge, no network meta-analysis has evaluated all available antiviral treatments for severe influenza. The optimal antiviral drug for treatment of patients with severe influenza remains uncertain.

**Added value of this study**

We found low certainty evidence that oseltamivir and peramivir might reduce the duration of hospitalisation in patients with severe seasonal influenza compared with placebo or standard care. Great uncertainty remains regarding the effects of oseltamivir, peramivir, and zanamivir on mortality in patients with severe seasonal influenza or zoonotic influenza. We identified no important differences in adverse events or serious adverse events associated with oseltamivir, peramivir, or zanamivir for treatment of patients with severe influenza, although the evidence is of very low certainty. The effects of other antivirals, including baloxavir, on mortality and other important outcomes in patients with severe influenza are very uncertain.

**Implications of all the available evidence**

Our study provides evidence that oseltamivir and peramivir, relative to placebo or standard care, might reduce the duration of hospitalisation for patients with severe seasonal influenza. These findings primarily highlight the uncertainty regarding effects of antivirals for treatment of patients with severe influenza but do provide some justification for their use. More clinical trials of antivirals are needed to inform the clinical benefit, safety, and effects on antiviral resistance in patients with severe influenza.

Antivirals, such as neuraminidase inhibitors, are recommended for and administered to patients with severe influenza.^14^ Systematic reviews and meta-analyses have reported that early neuraminidase inhibitor treatment could be associated with reduced mortality and shorter length of hospital stay compared with later or no neuraminidase inhibitor treatment for hospitalised influenza.^15^, ^16^, ^17^, ^18^, ^19^ However, these pairwise meta-analyses focused primarily on the relative effects of one class of antivirals (neuraminidase inhibitors) for treatment of severe seasonal or pandemic influenza and did not assess effects of antivirals on zoonotic influenza, nor assess the certainty of evidence.^15^, ^16^, ^17^, ^18^, ^19^ To our knowledge, no network meta-analysis has evaluated all available antiviral treatments for severe influenza. The optimal antiviral drug for treatment of hospitalised patients with influenza remains uncertain.

To support an update of the WHO clinical guidelines for influenza,^20^ we performed a systematic review and network meta-analysis of randomised controlled trials to assess the efficacy and safety of antivirals for severe influenza.

## Methods

### Search strategy and selection criteria

With the aid of a medical librarian, we searched MEDLINE, Embase, Cochrane Central Register of Controlled Trials, Cumulative Index to Nursing and Allied Health Literature, Global Health, Epistemonikos, and ClinicalTrials.gov from database inception up to Sept 20, 2023, and reviewed reference lists of relevant systematic reviews to identify additional trials. We used search strategies that combined controlled vocabulary (eg, Medical Subject Headings) and free-text terms. The search terms included “influenza”, “antiviral”, and “randomized controlled trials” (appendix pp 3–9).

Eligible randomised controlled trials enrolled hospitalised patients with suspected or laboratory-confirmed influenza (confirmed by RT-PCR assay, rapid antigen test, or immunofluorescence assay) and compared direct-acting antivirals against placebo, standard care without placebo, or another antiviral for treatment of severe influenza. Severe influenza was defined by WHO as an illness with laboratory-confirmed influenza that requires hospitalisation.^20^ We focused on antivirals approved for treatment of influenza by the US Food and Drug Administration or other regulatory organisations worldwide, including baloxavir, oseltamivir, laninamivir, zanamivir, peramivir, umifenovir, favipiravir, amantadine, and rimantadine.^21^ We did not apply restrictions on the type or subtype of influenza virus, publication language, patient age, or dose and administration route of antivirals. We excluded studies that investigated influenza prevention with vaccines, Chinese medicines, antivirals combined with adjunctive therapies, or antivirals used for pre-exposure or post-exposure chemoprophylaxis.

Using Covidence, pairs of reviewers (YG, ML, YZ, SL, and XC) independently screened titles and abstracts of all citations and full texts of potentially eligible records. We checked retractions for all eligible publications; if a study was retracted, we excluded the study from our review.^22^ Pairs of reviewers (YG, ML, YS, JX, QZ, ZL, and WZ) independently extracted data on study characteristics, patient characteristics, antiviral characteristics, and outcomes (appendix p 10). Reviewers resolved discrepancies by discussion or, if necessary, with the assistance of a third reviewer for adjudication.

We registered this systematic review protocol with PROSPERO (CRD42023456650) and reported the review according to the PRISMA guideline for network meta-analyses.^23^

### Data analysis

The independent WHO guideline panel identified important patient outcomes as follows: time to alleviation of symptoms, duration of hospitalisation, admission to intensive care unit (ICU), progression to invasive mechanical ventilation, duration of mechanical ventilation, mortality, hospital discharge destination, emergence of antiviral resistance, adverse events, adverse events related to treatments, and serious adverse events. We defined time to alleviation of symptoms as the time between the start of treatment and the point at which influenza-associated symptoms were alleviated.^24^, ^25^

Using the Hartung-Knapp-Sidik-Jonkman random-effects model, we conducted pairwise meta-analyses for each direct comparison. For dichotomous outcomes, we calculated risk ratios (RRs) with 95% CIs for mortality, progression to invasive mechanical ventilation, emergence of resistance, any adverse events, adverse events related to treatments, and serious adverse events, and we calculated risk differences with 95% CIs for ICU admission. For continuous outcomes, we calculated mean differences (MDs) with 95% CIs. When SDs were missing, we estimated them using the methods described in the Cochrane Handbook.^26^ To assess the between-study heterogeneity, we used the *I*^2^ statistic and visually inspected forest plots. For comparisons that included at least ten studies, to assess publication bias we planned to use Harbord's test for dichotomous outcomes and Egger's test for continuous outcomes,^27^, ^28^ as well as a visual assessment of the funnel plot.

We drew network plots for outcomes using Stata version 15.0. We conducted frequentist random-effects network meta-analyses employing a graph-theoretical approach, with the estimator derived from weighted least-square regression using the Moore-Penrose pseudoinverse method.^29^ Employing the design-by-treatment model (global test), we assessed the coherence assumption for the entire network.^30^ We calculated indirect estimates from the network by node-splitting and a back-calculation method.^31^ To assess local (loop-specific) incoherence within each closed loop of the network, measuring the difference between direct and indirect evidence, we applied the node-splitting method and computed a p value for the incoherence test.^32^ We conducted the analyses in R version 4.2.1.

To facilitate interpretation of results, we calculated absolute effects using RR estimates and the baseline risk estimates for outcomes in which the summary measure was RR. To estimate absolute effects of antivirals on mortality, the WHO guideline panel recommended use of two baseline risk categories for severe seasonal influenza and zoonotic influenza. We defined zoonotic influenza as novel influenza A viruses that are known to cause severe illness in infected humans, such as avian influenza A(H5N1), A(H5N6), and A(H7N9). We obtained baseline risks of mortality for severe seasonal influenza (30 per 1000 patients) and zoonotic influenza (387 per 1000 patients) from meta-analyses (results will be reported elsewhere). For other outcomes for which reliable observational data were not available, we used the median baseline risk in the control group of eligible randomised controlled trials.

If data were available (at least two trials providing relevant information for each subgroup), we planned to perform the following prespecified within-trial subgroup analyses for patients with severe influenza: (1) severe influenza aetiology: seasonal influenza A and B viruses versus zoonotic influenza A viruses versus pandemic influenza A viruses (hypothesis: antiviral treatment has lower effectiveness in patients with zoonotic influenza than in those with seasonal or pandemic influenza); (2) confirmed versus suspected influenza virus infection (hypothesis: reduced treatment effect in patients with suspected influenza *vs* patients with laboratory-confirmed influenza); and (3) age: infants (<2 years) versus children (2–12 years) versus adolescents and adults (13–64 years) versus older people (≥65 years; hypothesis: reduced treatment effect in older people). We planned to assess the credibility of significant subgroup effects using the Instrument to Assess the Credibility of Effect Modification Analyses tool.^33^

Pairs of reviewers independently evaluated the risk of bias of eligible randomised controlled trials using a modified Cochrane risk of bias tool (appendix p 11).^34^

We used the Grading of Recommendations Assessment, Development and Evaluation (GRADE) approach to assess certainty of evidence.^35^, ^36^ By considering the risk of bias, inconsistency, indirectness, imprecision, publication bias, intransitivity, and incoherence, we rated certainty of evidence for each comparison and outcome as high, moderate, low, or very low.^37^, ^38^ To assess intransitivity, we examined the distribution of potential effect modifiers, including age, influenza virus aetiology, and confirmed or suspected influenza, across treatment comparisons. We assessed imprecision at the network level using the minimally important difference (MID) for an outcome as a threshold.^39^ The WHO guideline panel specified an MID of 0·3% for mortality, 1·5% for progression to invasive mechanical ventilation, 1% for admission to the ICU, 1% for any adverse events and adverse events related to treatments, 0·5% for serious adverse events, 5% for emergence of antiviral resistance, and 1 day each for duration of hospitalisation, time to alleviation of symptoms, and duration of mechanical ventilation. We rated imprecision following GRADE guidance.^40^ If incoherence was present, we used the estimate with the higher certainty of direct and indirect evidence as the best estimate. We developed the summary of findings tables in MAGICapp following GRADE guidance.^41^, ^42^

### Role of the funding source

The funder of the study had no role in study design, data collection, data analysis, data interpretation, or writing of the report.

## Results

**Figure 1:**
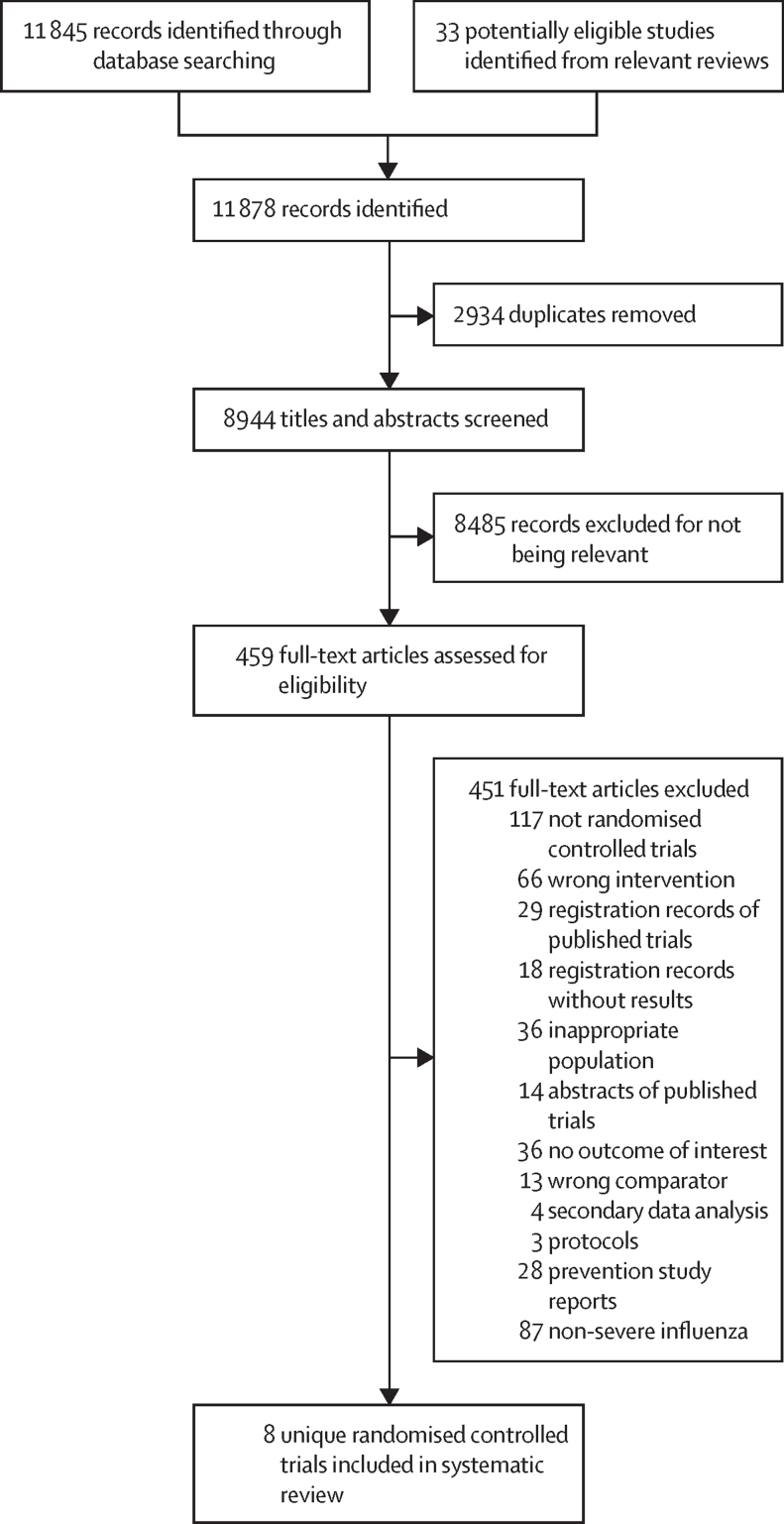
Study selection

Our search identified 11 878 citations, of which 8944 citations remained after removing duplicates. After screening 8944 titles and abstracts and 459 full texts, eight randomised controlled trials^43^, ^44^, ^45^, ^46^, ^47^, ^48^, ^49^, ^50^ were eligible for inclusion in this systematic review (figure 1).

The eight eligible trials included a total of 1424 participants (ranging from 30 to 615 per trial). The mean age ranged from 36 years to 60 years (for trials that reported mean or median age), the proportion of male patients ranged from 43% to 78%, and the proportion of patients with laboratory-confirmed influenza ranged from 79% to 100%. The interventions included oseltamivir, peramivir, zanamivir, rimantadine, zanamivir plus rimantadine, and baloxavir plus neuraminidase inhibitors. Direct comparisons between antivirals and standard care or placebo were available for oseltamivir and peramivir in three trials. Standard care was based on local institutional protocols or the primary physician's discretion, typically without the use of neuraminidase inhibitors. The other five trials compared different antivirals: two trials compared oseltamivir against peramivir, one compared oseltamivir against zanamivir, one compared zanamivir and rimantadine against rimantadine alone, and one trial compared baloxavir plus various neuraminidase inhibitors against neuraminidase inhibitors alone (table 1, appendix p 12).

**Table 1:** Basic characteristics of randomised controlled trials included in the systematic review

	**Country**	**Recruitment period**	**Patients randomly assigned, n**	Age, years, mean (SD)*	**Age range, years**	**Proportion of male patients, %**	**Baseline type of influenza**	**Confirmed influenza, %**	**Treatments**	**Outcomes**
Chen et al (2020)	China	December, 2018 to April, 2019	40	36 (18)	18–85	43%	5% A(H1N1), 95% A(H3N2)	100%	Oseltamivir oral 75 mg twice daily for 5 days *vs* peramivir intravenous 300 mg once daily for 5 days	Time to alleviation of symptoms
Dawood et al (2016)	EI Salvador, Panama	September to October, 2012; April to October, 2013	30	NR	0–9	NR	17% A(H1N1), 70% A(H3N2), 13% B	100%	Oseltamivir every 12 h for 10 doses; for children aged 0–11 months, study drug was dosed at 3 mg/kg per dose; for children aged 12 months, study drug was dosed based on standard unit dosing: 30 mg per dose for children ≤15 kg, 45 mg for children >15 kg to 23 kg, 60 mg for children >23 kg to 40 kg, and 75 mg for children >40 kg; comparator group received placebo	Duration of hospitalisation
de Jong et al (2014)	21 countries	September, 2009 to November, 2012	121	43 (NR)	13–86	53%	50% A(H3N2), 21% A(2009 H1N1), 3% A(indeterminate), 24% B, 2% A and B	100%	Peramivir intravenous 600 mg once daily for 5 days; comparator group received standard care	Mortality, admission to ICU, and time to alleviation of symptoms
Ison et al (2003)	USA	January, 1998 to April, 1999	41	59 (17)	22–93	78%	93% A, 2% B (remaining patients were negative for influenza)	95%	Zanamivir (16 mg by inhalation four times a day for 5 days) plus rimantadine (orally for 5 days); the dose of rimantadine was 100 mg twice daily for patients aged 10–64 years, or 100 mg once daily for patients with severe hepatic dysfunction or renal failure (creatinine clearance ≤10 mL/min) and patients aged ≥65 years; comparator group received rimantadine orally for 5 days	Mortality, any adverse events, serious adverse events, and duration of hospitalisation
Ison et al (2013)	Australia, Canada, China, New Zealand, South Africa, USA	July, 2007 to September, 2008	137	59 (22)	≥18	47%	56% A(H3N2), 18% A(H1N1), 26% B	100%	Peramivir 200 mg intravenously once daily for 5 days *vs* peramivir 400 mg intravenously once daily for 5 days *vs* oseltamivir oral 75 mg twice daily for 5 days	Mortality, admission to ICU, any adverse events, serious adverse events, duration of hospitalisation, and time to alleviation of symptoms
Kumar et al (2022)	25 countries	January, 2019 to March, 2020	366	60 (20)	12–96	53%	50% A(H1N1), 37% A(H3N2), 8% B, 1% multiple subtypes, 4% unknown	100%	Baloxavir plus NAIs; baloxavir was given enterally at 40 mg (for bodyweight 40 kg to <80 kg) or 80 mg (for ≥80 kg) on day 1 and day 4, with an additional dose on day 7 if no clinical improvement had occurred on day 5; comparator group received standard-of-care NAIs (oseltamivir, zanamivir, or peramivir), administered according to local clinical practice	Mortality, admission to ICU, progression to mechanical ventilation, emergence of resistance, adverse events related to antivirals, any adverse events, serious adverse events, duration of mechanical ventilation, and duration of hospitalisation
Marty et al (2017)	26 countries	January, 2011 to February, 2015	615	57 (NR)	15–101	54%	36% A(H3N2), 30% A(H1N1)pdm09, 11% B, <1% A(H1N1)pdm09 and A(H3N2), <1% A(H1N1)pdm09 and B, 1% A(H3N2) and B, <1% untyped A and B (remaining patients had suspected influenza)	79%	Zanamivir intravenous 300 mg twice a day for 5–10 days *vs* zanamivir intravenous 600 mg twice a day for 5–10 days *vs* oseltamivir oral 75 mg twice daily for 5–10 days	Mortality, progression to mechanical ventilation, emergence of resistance, adverse events related to antivirals, any adverse events, serious adverse events, and duration of mechanical ventilation
Ramirez et al (2018)	USA	2010 to 2013	74	NR	≥18	NR	NR	100%	Oseltamivir oral 75 mg twice daily for 7 days *vs* standard care	Mortality and duration of hospitalisation

NR=not reported. ICU=intensive care unit. NAI=neuraminidase inhibitor.

* If the mean (SD) was not available, the median age and related statistics were used to calculate the mean (SD) during data extraction.

The risk of bias of eligible trials for each outcome is presented in the appendix (pp 13–15). Most biases were due to inadequate allocation concealment and lack of blinding. We rated one trial as having a low or probably low risk of bias for all reported outcomes.^48^

**Figure 2:**
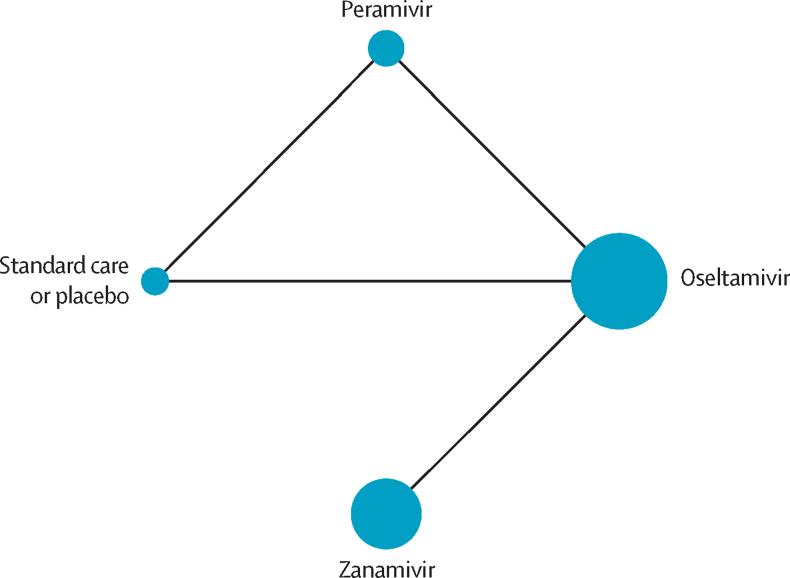
Network plot for mortality The size of the circle represents the number of participants. The connecting lines represent direct comparisons. The width of the line represents the number of studies.

Six trials were included in the network meta-analysis.^43^, ^44^, ^45^, ^47^, ^49^, ^50^ Network plots for each outcome are presented in figure 2 and the appendix (pp 16–20). We did not find substantial between-study heterogeneity (appendix p 21), global incoherence (appendix p 22), or local incoherence (appendix pp 23–28). The GRADE summary of findings is presented in Table 2, Table 3 and the appendix (pp 29–34). We judged the certainty of evidence to be low or very low for all outcomes. We did not include two eligible trials in the network meta-analysis because both arms of these two trials did not connect with other interventions in the network.^46^, ^48^

**Table 2:** GRADE summary of findings for mortality for different comparisons

	**Study results and measurements**	**Seasonal influenza**				**Zoonotic influenza**			
		Absolute effect estimate per 1000 patients	Absolute difference per 1000 patients (95% CI)	Certainty in effect estimates	Summary	Absolute effect estimate per 1000 patients	Absolute difference per 1000 patients (95% CI)	Certainty in effect estimates	Summary
Oseltamivir *vs* standard care or placebo	RR 0·53 (95% CI 0·07 to 4·24); based on data from 74 participants in one study	16 *vs* 30	−14 (−28 to 97)	Very low*	Whether oseltamivir reduces mortality in people with seasonal influenza is very uncertain	205 *vs* 387	−182 (−360 to 613)	Very low*	Whether oseltamivir reduces mortality in people with zoonotic influenza is very uncertain
Peramivir *vs* standard care or placebo	RR 0·40 (95% CI 0·03 to 4·72); based on data from 114 participants in one study	12 *vs* 30	−18 (−29 to 112)	Very low*†	Whether peramivir reduces mortality in people with seasonal influenza is very uncertain	155 *vs* 387	−232 (−375 to 613)	Very low*†	Whether peramivir reduces mortality in people with zoonotic influenza is very uncertain
Zanamivir *vs* standard care or placebo	RR 0·58 (95% CI 0·06 to 5·29); based on indirect evidence	17 *vs* 30	−13 (−28 to 129)	Very low*†	Whether zanamivir reduces mortality in people with seasonal influenza is very uncertain	224 *vs* 387	−163 (−364 to 613)	Very low*†	Whether zanamivir reduces mortality in people with zoonotic influenza is very uncertain
Oseltamivir *vs* peramivir	RR 1·33 (95% CI 0·11 to 15·87); based on data from 137 participants in one study	16 *vs* 12	4 (−11 to 178)	Very low*	Whether oseltamivir reduces mortality in people with seasonal influenza compared with peramivir is very uncertain	206 *vs* 155	51 (−138 to 845)	Very low*	Whether oseltamivir reduces mortality in people with zoonotic influenza compared with peramivir is very uncertain
Oseltamivir *vs* zanamivir	RR 0·91 (95% CI 0·44 to 1·87); based on data from 488 participants in one study	15 *vs* 17	−2 (−10 to 15)	Very low†‡	Whether oseltamivir reduces mortality in people with seasonal influenza compared with zanamivir is very uncertain	204 *vs* 224	−20 (−126 to 195)	Very low*†	Whether oseltamivir reduces mortality in people with zoonotic influenza compared with zanamivir is very uncertain
Peramivir *vs* zanamivir	RR 0·68 (95% CI 0·05 to 9·01); based on indirect evidence	11 *vs* 17	−6 (−17 to 139)	Very low*†	Whether peramivir reduces mortality in people with seasonal influenza compared with zanamivir is very uncertain	152 *vs* 224	−72 (−213 to 776)	Very low*†	Whether peramivir reduces mortality in people with zoonotic influenza compared with zanamivir is very uncertain

GRADE=Grading of Recommendations Assessment, Development and Evaluation. RR=risk ratio.

* Rating decreased by three levels for imprecision.

† Rating decreased by one level for risk of bias.

‡ Rating decreased by two levels for imprecision.

**Table 3:** GRADE summary of findings for duration of hospitalisation for different comparisons

	**Study results and measurements**	**Absolute effect estimates, mean duration in days**	**Mean difference (95% CI)**	**Certainty in effect estimates**	**Summary**
Oseltamivir *vs* standard care or placebo	The lower the duration of hospitalisation, the better the result; based on data from 104 participants in two studies	3·37 *vs* 5·00	−1·63 (−2·81 to −0·45)	Low*†	Oseltamivir might reduce duration of hospitalisation
Peramivir *vs* standard care or placebo	The lower the duration of hospitalisation, the better the result; based on indirect evidence	3·27 *vs* 5·00	−1·73 (−3·33 to −0·13)	Low*†	Peramivir might reduce duration of hospitalisation
Oseltamivir *vs* peramivir	The lower the duration of hospitalisation, the better the result; based on data from 122 participants in one study	3·37 *vs* 3·27	0·10 (−0·98 to 1·18)	Low*†	There might be little or no difference between oseltamivir and peramivir for duration of hospitalisation

GRADE=Grading of Recommendations Assessment, Development and Evaluation.

* Rating decreased by one level for risk of bias.

† Rating decreased by one level for imprecision.

The network meta-analysis of mortality included four trials of oseltamivir, peramivir, or zanamivir, involving 813 patients with severe seasonal influenza.^45^, ^47^, ^49^, ^50^ The risk differences for the effect of oseltamivir, peramivir, or zanamivir on mortality when compared with placebo or standard care, or with each other, varied from 18 fewer to four more per 1000 patients for seasonal influenza and from 232 fewer to 51 more per 1000 patients for zoonotic influenza (very low certainty evidence; table 2).

The network meta-analysis of ICU admission included two trials of oseltamivir or peramivir among 235 patients with severe seasonal influenza.^45^, ^47^ The risk differences for the effect of oseltamivir or peramivir on ICU admission compared with standard care, or with each other, varied from 29 fewer to 43 more per 1000 patients (very low certainty evidence; appendix p 29).

The network meta-analysis of duration of hospitalisation included three trials of oseltamivir or peramivir, involving 226 patients with severe seasonal influenza.^44^, ^47^, ^50^ The MD in hospitalisation duration for oseltamivir compared with standard care or placebo was 1·63 days shorter (95% CI –2·81 to –0·45; low certainty evidence). The MD for peramivir compared with placebo or standard care was 1·73 days shorter (–3·33 to –0·13; low certainty evidence). The MD in hospitalisation duration for oseltamivir compared with peramivir was 0·10 days longer (–0·98 to 1·18; low certainty evidence; table 3).

The network meta-analysis of time to alleviation of symptoms included three trials that assessed the effect of oseltamivir or peramivir, involving 283 patients with severe seasonal influenza.^43^, ^45^, ^47^ The MD in time to alleviation of symptoms for oseltamivir compared with standard care was 0·34 days longer (95% CI –0·86 to 1·54 days; low certainty evidence). The MD in time to alleviation of symptoms for peramivir compared with standard care was 0·05 days shorter (–0·69 to 0·59 days; low certainty evidence; appendix p 30).

Two trials with 752 patients with severe influenza provided data on any adverse events and serious adverse events comparing oseltamivir, peramivir, and zanamivir.^47^, ^49^ There were no convincing differences in any adverse events or serious adverse events among the three antivirals (very low certainty evidence; appendix pp 31–32).

Only one trial^49^ reported data on progression to mechanical ventilation, duration of mechanical ventilation, emergence of resistance, and adverse events related to oseltamivir or zanamivir treatment. Available data did not permit conducting network meta-analyses for these outcomes but pairwise meta-analyses were possible. Compared with zanamivir, the RRs of oseltamivir for progression to mechanical ventilation, emergence of antiviral resistance, or adverse events related to treatment ranged from 1·20 to 2·89 with 95% CIs overlapping with the null effect (very low certainty evidence; appendix p 33). The MD in duration of mechanical ventilation was 0·89 days (95% CI –2·32 to 4·10; very low certainty evidence; appendix p 34). None of the included trials assessed hospital discharge destination.

One study investigated combination treatment with baloxavir plus neuraminidase inhibitors versus monotherapy with neuraminidase inhibitors.^48^ There were few or no differences with the addition of baloxavir in terms of duration of hospitalisation (MD 0·31 days shorter, 95% CI –0·73 to 0·11; low certainty evidence) or emergence of antiviral resistance (risk difference 25 fewer per 1000 patients, 95% CI –39 to 42; low certainty evidence). Very low certainty evidence was available on the effects of baloxavir plus neuraminidase inhibitors on ICU admission, mechanical ventilation, mortality, or adverse events compared with neuraminidase inhibitors alone (appendix pp 35–37).

One study compared zanamivir plus rimantadine with rimantadine alone.^46^ Very low certainty evidence was available on the effects of zanamivir plus rimantadine on duration of hospitalisation, mortality, or adverse events compared with rimantadine monotherapy (appendix pp 38–39).

Due to the small number of eligible trials, we could not perform planned subgroup analyses or test for publication bias.

## Discussion

In this systematic review and network meta-analysis, we found that oseltamivir and peramivir might reduce duration of hospitalisation in patients with severe seasonal influenza compared with placebo or standard care, but the evidence was of low certainty due to scarce data from the small number of included randomised controlled trials. The effects of oseltamivir, peramivir, or zanamivir on mortality in patients with severe seasonal influenza or severe zoonotic influenza compared with placebo or standard care are very uncertain. Uncertainty also remains about the effects of oseltamivir, peramivir, and zanamivir on ICU admission. We did not find evidence of differences in any adverse events or serious adverse events among oseltamivir, peramivir, and zanamivir.

This is the first systematic review and network meta-analysis to evaluate the efficacy and safety of different antivirals for treatment of severe influenza. We focused on evidence for approved antivirals from randomised controlled trials, assessed the certainty of evidence using the GRADE approach, and presented absolute effects for outcomes. To reflect typical clinical scenarios in practice, we used two separate baseline risks for mortality and separately estimated absolute effects for severe seasonal influenza and zoonotic influenza. The selection of patient-important outcomes, baseline risks, and MID values for outcomes was based on the independent WHO guideline panel's discussions and suggestions. The WHO panel also reviewed the results and assisted in their interpretation, ensuring a consistent interpretation of the available evidence to date. This systematic review provides the evidence base for the WHO clinical guideline recommendations for antiviral treatment of severe influenza.

Our review has limitations. First, only eight eligible trials were identified, and six trials were included in the network meta-analyses. Only one trial that compared oseltamivir to zanamivir provided data on progression to mechanical ventilation, duration of mechanical ventilation, emergence of resistance, and adverse events related to antiviral treatment.^49^ No trials addressed the effects of antivirals versus placebo or standard care on any adverse events or serious adverse events. Therefore, uncertainty remains about the effects of antivirals on most outcomes for patients with severe influenza. Second, due to sparse data available, we were unable to perform any prespecified subgroup analyses or assess the impact of secondary bacterial infection and influenza type (A or B) on outcomes. Similarly, the assessments of incoherence and heterogeneity were not applicable for most outcomes and evaluation of publication bias was not applicable for all outcomes. Third, because the mean age of the patients in eligible randomised controlled trials ranged from 36 years to 60 years, data were scarce on the effect of antivirals on individuals older than 60 years and for children. The effects of antivirals in children and older adults with severe influenza have not been conclusively addressed by specific randomised controlled trials or subgroup analyses. Fourth, the WHO guideline panel suggested estimating separate absolute effects of antivirals on mortality for hospitalised patients with seasonal influenza and for zoonotic influenza. Because nearly all participants included in the eligible trials were patients with severe seasonal influenza, we estimated the absolute effects for patients with severe zoonotic influenza using the network relative estimates for severe seasonal influenza and baseline risk from a meta-analysis. Fifth, some trials were at risk of bias due to inadequate allocation concealment, lack of blinding, or incomplete outcome data. These issues warrant greater attention in future randomised controlled trials of antiviral treatment of patients with severe influenza. Due to decreasing the rating of available evidence for risk of bias and imprecision, the certainty of evidence was assessed to be low or very low for all available comparisons and outcomes. If new data from randomised controlled trials become available (eg, NCT02735707 and NCT04381936), we anticipate that the certainty of evidence will improve. To provide up-to-date evidence, we will periodically update this systematic review.

One previous pairwise meta-analysis of 90 studies (all observational studies) of antiviral treatment of hospitalised patients with pandemic influenza A(H1N1)pdm09 virus infection reported that neuraminidase inhibitor treatment at any time versus none was associated with a non-significant reduction in mortality, but early neuraminidase inhibitor treatment (≤48 h after symptom onset) versus late, and early antiviral treatment initiation versus none, were associated with significant reductions in mortality.^15^ Another individual participant data meta-analysis that included 29 234 hospitalised patients with pandemic influenza A(H1N1)pdm09 virus infection from 78 observational studies reported that neuraminidase inhibitor treatment (irrespective of timing) was associated with a reduction in mortality compared with no treatment, and early treatment (within 2 days of symptom onset) was associated with a reduction in mortality compared with later treatment or no treatment.^16^ These meta-analyses reported inconsistent results regarding the effect of neuraminidase inhibitor treatment of patients with severe influenza at any time versus no neuraminidase inhibitor treatment on mortality, mainly because of the different kinds of data used (aggregate data *vs* individual participant data).

Our network meta-analysis, including only randomised controlled trials, did not substantiate the findings of previous meta-analyses. We assessed the effect of each antiviral on patient-important outcomes and presented absolute effects for mortality in patients with severe seasonal influenza and estimated absolute effects for mortality in patients with severe zoonotic influenza, although the very low certainty evidence indicated low confidence in inferences regarding mortality. Moreover, because all included trials did not present data related to the timing of antiviral treatment initiation in relation to symptom onset, we were unable to examine the effect of the timing of antiviral treatment initiation from symptom onset on outcomes.

One pairwise meta-analysis that included seven randomised controlled trials addressed different dosages and regimens of neuraminidase inhibitors in hospitalised patients with seasonal or pandemic influenza, and reported non-significant differences among different antiviral treatment regimens in terms of mortality, time to clinical resolution, and viral clearance.^18^ These findings regarding time to clinical resolution are consistent with our results of antivirals not having important effects on reducing time to alleviation of symptoms in patients with severe influenza.

One previous individual participant data meta-analysis that included observational studies of patients hospitalised with pandemic influenza A(H1N1)pdm09 virus infection reported that neuraminidase inhibitor treatment started on the day of admission, regardless of time since symptom onset, was associated with a reduction in the length of hospital stay compared with no or later initiation of neuraminidase inhibitor treatment.^19^ Our meta-analysis also found that oseltamivir and peramivir might reduce the duration of hospitalisation in patients with severe influenza compared with placebo or standard care. The WHO guideline panel discussed the evidence from observational studies and deemed that they did not provide a higher certainty of evidence for this population compared with the current systematic review of randomised controlled trials.

Due to limited data from the small number of randomised controlled trials of antivirals for treatment of patients with severe seasonal influenza and a lack of randomised controlled trials for treatment of severe zoonotic influenza, the current level of evidence for antiviral treatment of severe seasonal or zoonotic influenza is of low certainty. Additional clinical trials of antivirals are needed to inform the clinical benefit, safety, and effects on antiviral resistance in patients with severe influenza. Important gaps include better evidence on the effects of antiviral treatment for patients with severe influenza on admission to ICU, progression to invasive mechanical ventilation, duration of mechanical ventilation, mortality, and emergence of antiviral resistance, and the effects of antivirals on outcomes in key subgroup populations, including patients with severe zoonotic influenza.

Data from randomised controlled trials of antiviral treatment for patients with severe influenza are scarce. In patients with severe influenza, oseltamivir or peramivir might reduce the duration of hospitalisation compared with placebo or standard care. There is high uncertainty regarding the effects of oseltamivir, peramivir, and zanamivir on ICU admission and mortality in patients with severe seasonal or zoonotic influenza. Sufficiently powered clinical trials in patients with severe influenza due to seasonal influenza virus infections and novel influenza A virus infections are needed to provide higher certainty evidence of the effects of antiviral treatment on important clinical outcomes.

## Contributors

## Data sharing

Data in this systematic review and meta-analysis are extracted from published studies available elsewhere. All processed data are presented in this Article and the appendix.

## Declaration of interests

We declare no competing interests.
