# Supplementary material for: Antivirals for treatment of severe influenza: a systematic review and network meta-analysis of randomised controlled trials
Source: Lancet. 2024 Aug 24;404(10454):753–63. doi: 10.1016/S0140-6736(24)01307-2 (PMC11369965; doi:10.1016/S0140-6736(24)01307-2)
Supplement: Supplementary appendix [file mmc1.pdf]

THE LANCET

Supplementary appendix

This appendix formed part of the original submission and has been peer reviewed. We post it as supplied by the authors.

Supplement to: Gao Y, Guyatt G, Uyeki TM, et al. Antivirals for treatment of severe influenza: a systematic review and network meta-analysis of randomised controlled trials. *Lancet* 2024; **404:** 753–63.

Antivirals for treatment of severe influenza: a systematic review and network meta-analysis of randomized controlled trials

Contents

Appendix 1. Search strategy for databases 3
Appendix 2. Details of methods 10
2.1. Details of data extraction 10
2.2. Details of risk of bias assessment 11
Appendix 3. Additional characteristics of eligible RCTs 12
Appendix 4. Risk of bias for eligible studies 13
Appendix 5. Network plots 16
5.1. Network plot for admission to ICU 16
5.2. Network plot for duration of hospitalization 17
5.3. Network plot for time to alleviation of symptoms 18
5.4. Network plot for any adverse events 19
5.5. Network plot for serious adverse events 20
Appendix 6. Assessment of between-study heterogeneity 21
Appendix 7. Assessment of global incoherence 22
Appendix 8. Direct, indirect, and network treatment estimates 23
8.1. Direct, indirect, and network treatment estimates for mortality 23
8.2. Direct, indirect, and network treatment estimates for admission to ICU 24
8.3. Direct, indirect, and network treatment estimates for duration of hospitalization 25
8.4. Direct, indirect, and network treatment estimates for time to alleviation of symptoms 26
8.5. Direct, indirect, and network treatment estimates for any adverse events 27
8.6. Direct, indirect, and network treatment estimates for serious adverse events 28
Appendix 9. GRADE summary of findings for outcomes 29
9.1. GRADE summary of findings for admission to ICU for different comparisons 29
9.2. GRADE summary of findings for time to alleviation of symptoms for different comparisons 30

9.3. GRADE summary of findings for any adverse events for different comparisons 31
9.4. GRADE summary of findings for serious adverse events for different comparisons ... 32
9.5. GRADE summary of findings for progression to mechanical ventilation, emergence of resistance, and adverse events related to treatments 33
9.6. GRADE summary of findings for duration of mechanical ventilation 34
Appendix 10. Results of a study, comparing baloxavir plus NAIs with NAIs, not included in the network meta-analysis 35
10.1. Forest plots for baloxavir plus NAIs versus NAIs (oseltamivir, zanamivir, or peramivir) 35
10.2. GRADE summary of findings for baloxavir plus NAIs versus NAIs (oseltamivir, zanamivir, or peramivir) 36
Appendix 11. Results of a study, comparing zanamivir plus rimantadine with rimantadine, not included in the network meta-analysis.. 38
11.1. Forest plots for zanamivir plus rimantadine versus rimantadine 38
11.2. GRADE summary of findings for zanamivir plus rimantadine versus rimantadine 39

Appendix 1. Search strategy for databases

Ovid MEDLINE(R) ALL

1 exp Influenza, Human/
2 exp Influenza A virus/
3 exp Influenza B virus/
4 exp Influenzavirus C/
5 (Influenza or flu or H1N1 or PH1N1 or H3N2 or AH1N1 or AH3N2 or H5N1 or H7N9).mp. [mp=title, book title, abstract, original title, name of substance word, subject heading word, floating sub-heading word, keyword heading word, organism supplementary concept word, protocol supplementary concept word, rare disease supplementary concept word, unique identifier, synonyms, population supplementary concept word, anatomy supplementary concept word]
6 or/1-5
7 Antiviral agents/
8 Antiviral*.tw.
9 (neuraminidase inhibitor* or NA inhibitor*).tw.
10 Oseltamivir/ or Zanamivir/
11 (oseltamivir or tamiflu or "GS 4104" or GS4104 or GS-4104 or "GS 4071" or GS4071 or GS-4071 or zanamivir or relenza or "GG 167" or GG167 or GG-167 or CS-8958 or Dectova or Laninamivir or R-125489 or R125489 or "R 125489" or Inavir or peramivir or "BCX 1812" or BCX1812 or BCX-1812 or "RWJ 270201" or RWJ270201 or RWJ-270201 or Rapivab or rapiacta).ti,ab.
12 Viral Polymerase Complex Inhibitor*.tw.
13 (Favipiravir or T-705 or Avigan or FabiFlu or Pimodivir or VX-787 or JNJ-63623872 or AL-794 or ALS-033719 or ZSP1273 or Enisamium iodide or FAV00A or TG-1000 or GP681).ti,ab.
14 matrix protein 2 ion channel inhibitor*.tw.
15 (Radavirsen or AVI-7100).ti,ab.
16 cap-dependent endonuclease inhibitor*.tw.
17 ("Baloxavir marboxil" or Baloxavir or S-033188 or Xofluza).ti,ab.
18 (Umifenovir or Arbidol or Arbidole).ti,ab.
19 Amantadine/ or Rimantadine/
20 (Amantadine or Symmetrel or Symetrel or Rimantadine or Flumadine or Roflual).ti,ab.
21 or/7-20
22 6 and 21
23 randomized controlled trial.pt.
24 controlled clinical trial.pt.
25 randomized.ab.
26 placebo.ab.
27 drug therapy.fs.
28 randomly.ab.
29 trial.ti.
30 groups.ab.
31 or/23-30

32 (animals not (humans and animals)).sh.
33 31 not 32
34 22 and 33

Ovid Embase

1 exp Influenza/ or Influenza virus/
2 exp Influenza A virus/ or exp Influenza A virus/
3 exp Influenza B/ or exp Influenza B virus/
4 exp Influenza C/ or exp Influenza C virus/
5 (Influenza or flu or H1N1 or PH1N1 or H3N2 or AH1N1 or AH3N2 or H5N1 or H7N9).mp. [mp=title, abstract, heading word, drug trade name, original title, device manufacturer, drug manufacturer, device trade name, keyword heading word, floating subheading word, candidate term word]
6 or/1-5
7 Antivirus agent/
8 Antiviral*.tw.
9 (neuraminidase inhibitor* or NA inhibitor*).tw.
10 Sialidase inhibitor/ or Oseltamivir/ or Zanamivir/ or Laninamivir/ or Peramivir/
11 (oseltamivir or tamiflu or "GS 4104" or GS4104 or GS-4104 or "GS 4071" or GS4071 or GS-4071 or zanamivir or relenza or "GG 167" or GG167 or GG-167 or CS-8958 or Dectova or Laninamivir or R-125489 or R125489 or "R 125489" or Inavir or peramivir or "BCX 1812" or BCX1812 or BCX-1812 or "RWJ 270201" or RWJ270201 or RWJ-270201 or Rapivab or rapiacta).ti,ab.
12 Viral Polymerase Complex Inhibitor*.tw.
13 Favipiravir/ or Pimodivir/ or (Favipiravir or T-705 or Avigan or FabiFlu or Pimodivir or VX-787 or JNJ-63623872 or AL-794 or ALS-033719 or ZSP1273 or Enisamium iodide or FAV00A or TG-1000 or GP681).ti,ab.
14 matrix protein 2 ion channel inhibitor*.tw.
15 Radavirsen/ or (Radavirsen or AVI-7100).ti,ab.
16 cap-dependent endonuclease inhibitor*.tw.
17 Baloxavir marboxil/ or ("Baloxavir marboxil" or Baloxavir or S-033188 or Xofluza).ti,ab.
18 Umifenovir/ or (Umifenovir or Arbidol or Arbidole).ti,ab.
19 Amantadine/ or Rimantadine/
20 (Amantadine or Symmetrel or Symetrel or Rimantadine or Flumadine or Roflual).ti,ab.
21 or/7-20
22 6 and 21
23 Randomized controlled trial/
24 Controlled clinical study/
25 random$.ti,ab.
26 randomization/
27 intermethod comparison/
28 placebo.ti,ab.

29 (compare or compared or comparison).ti.
30 ((evaluated or evaluate or evaluating or assessed or assess) and (compare or compared or comparing or comparison)).ab.
31 (open adj label).ti,ab.
32 ((double or single or doubly or singly) adj (blind or blinded or blindly)).ti,ab.
33 double blind procedure/
34 parallel group$1.ti,ab.
35 (crossover or cross over).ti,ab.
36 ((assign$ or match or matched or allocation) adj5 (alternate or group$1 or intervention$1 or patient$1 or subject$1 or participant$1)).ti,ab.
37 (assigned or allocated).ti,ab.
38 (controlled adj7 (study or design or trial)).ti,ab.
39 (volunteer or volunteers).ti,ab.
40 human experiment/
41 trial.ti.
42 or/23-41
43 (random$ adj sampl$ adj7 ("cross section$" or questionnaire$1 or survey$ or database$1)).ti,ab. not (comparative study/ or controlled study/ or randomi?ed controlled.ti,ab. or randomly assigned.ti,ab.)
44 Cross-sectional study/ not (randomized controlled trial/ or controlled clinical study/ or controlled study/ or randomi?ed controlled.ti,ab. or control group$1.ti,ab.)
45 (((case adj control$) and random$) not randomi?ed controlled).ti,ab.
46 (Systematic review not (trial or study)).ti.
47 (nonrandom$ not random$).ti,ab.
48 "Random field$".ti,ab.
49 (random cluster adj3 sampl$).ti,ab.
50 (review.ab. and review.pt.) not trial.ti.
51 "we searched".ab. and (review.ti. or review.pt.)
52 "update review".ab.
53 (databases adj4 searched).ab.
54 (rat or rats or mouse or mice or swine or porcine or murine or sheep or lambs or pigs or piglets or rabbit or rabbits or cat or cats or dog or dogs or cattle or bovine or monkey or monkeys or trout or marmoset$1).ti. and animal experiment/
55 Animal experiment/ not (human experiment/ or human/)
56 or/43-55
57 42 not 56
58 22 and 57

Cochrane Central Register of Controlled Trials

1 exp Influenza, Human/
2 exp Influenza A virus/
3 exp Influenza B virus/

4 exp Influenzavirus C/
5 (Influenza or flu or H1N1 or PH1N1 or H3N2 or AH1N1 or AH3N2 or H5N1 or H7N9).mp. [mp=title, original title, abstract, floating sub-heading word, mesh headings, heading words, keyword]
6 or/1-5
7 Antiviral agents/
8 Antiviral*.tw.
9 (neuraminidase inhibitor* or NA inhibitor*).tw.
10 Oseltamivir/ or Zanamivir/
11 (oseltamivir or tamiflu or "GS 4104" or GS4104 or GS-4104 or "GS 4071" or GS4071 or GS-4071 or zanamivir or relenza or "GG 167" or GG167 or GG-167 or CS-8958 or Dectova or Laninamivir or R-125489 or R125489 or "R 125489" or Inavir or peramivir or "BCX 1812" or BCX1812 or BCX-1812 or "RWJ 270201" or RWJ270201 or RWJ-270201 or Rapivab or rapiacta).ti,ab.
12 Viral Polymerase Complex Inhibitor*.tw.
13 (Favipiravir or T-705 or Avigan or FabiFlu or Pimodivir or VX-787 or JNJ-63623872 or AL-794 or ALS-033719 or ZSP1273 or Enisamium iodide or FAV00A or TG-1000 or GP681).ti,ab.
14 matrix protein 2 ion channel inhibitor*.tw.
15 (Radavirsen or AVI-7100).ti,ab.
16 cap-dependent endonuclease inhibitor*.tw.
17 ("Baloxavir marboxil" or Baloxavir or S-033188 or Xofluza).ti,ab.
18 (Umifenovir or Arbidol or Arbidole).ti,ab.
19 Amantadine/ or Rimantadine/
20 (Amantadine or Symmetrel or Symetrel or Rimantadine or Flumadine or Roflual).ti,ab.
21 or/7-20
22 6 and 21
23 randomized controlled trial.pt.
24 controlled clinical trial.pt.
25 randomized.ab.
26 placebo.ab.
27 drug therapy.fs.
28 randomly.ab.
29 trial.ti.
30 groups.ab.
31 or/23-30
32 (animals not (humans and animals)).sh.
33 31 not 32
34 22 and 33

Global Health

1 exp Influenza/ or Influenza viruses/
2 exp Influenza A virus/ or exp Influenza A virus/

3 exp Influenza B/ or exp Influenza B virus/
4 exp Influenza C/ or exp Influenza C virus/
5 (Influenza or flu or H1N1 or PH1N1 or H3N2 or AH1N1 or AH3N2 or H5N1 or H7N9).mp. [mp=abstract, title, original title, heading words, cabicodes words]
6 or/1-5
7 Antiviral agents/
8 Antiviral*.tw.
9 (neuraminidase inhibitor* or NA inhibitor*).tw.
10 Sialidase inhibitors/ or Oseltamivir/ or Zanamivir/ or Laninamivir/ or Peramivir/
11 (oseltamivir or tamiflu or "GS 4104" or GS4104 or GS-4104 or "GS 4071" or GS4071 or GS-4071 or zanamivir or relenza or "GG 167" or GG167 or GG-167 or CS-8958 or Dectova or Laninamivir or R-125489 or R125489 or "R 125489" or Inavir or peramivir or "BCX 1812" or BCX1812 or BCX-1812 or "RWJ 270201" or RWJ270201 or RWJ-270201 or Rapivab or rapiacta).ti,ab.
12 Viral Polymerase Complex Inhibitor*.tw.
13 Favipiravir/ or (Favipiravir or T-705 or Avigan or FabiFlu or Pimodivir or VX-787 or JNJ-63623872 or AL-794 or ALS-033719 or ZSP1273 or Enisamium iodide or FAV00A or TG-1000 or GP681).ti,ab.
14 matrix protein 2 ion channel inhibitor*.tw.
15 (Radavirsen or AVI-7100).ti,ab.
16 cap-dependent endonuclease inhibitor*.tw.
17 ("Baloxavir marboxil" or Baloxavir or S-033188 or Xofluza).ti,ab.
18 (Umifenovir or Arbidol or Arbidole).ti,ab.
19 Amantadine/ or Rimantadine/
20 (Amantadine or Symmetrel or Symetrel or Rimantadine or Flumadine or Roflual).ti,ab.
21 or/7-20
22 6 and 21
23 exp randomized controlled trials/
24 (randomized controlled trial or random* or blind* or placebo*).mp. [mp=abstract, title, original title, heading words, cabicodes words]
25 23 or 24
26 22 and 25

CINAHL

#	Query
S36	S23 AND S26 AND S35
S35	S27 OR S28 OR S29 OR S30 OR S31 OR S32 OR S33 OR S34
S34	TI (matrix protein 2 ion channel inhibitor* OR Radavirsen or AVI-7100 OR cap-dependent endonuclease inhibitor* OR "Baloxavir marboxil" or Baloxavir or S-033188 or Xofluza OR Umifenovir or Arbidol or Arbidole OR Amantadine or Symmetrel or

	Symetrel or Rimantadine or Flumadine or Roflual) OR AB (matrix protein 2 ion channel inhibitor* OR Radavirsen or AVI-7100 OR cap-dependent endonuclease inhibitor* OR "Baloxavir marboxil" or Baloxavir or S-033188 or Xofluza OR Umifenovir or Arbidol or Arbidole OR Amantadine or Symmetrel or Symetrel or Rimantadine or Flumadine or Roflual)
S33	(MH "Amantadine")
S32	TI (Viral Polymerase Complex Inhibitor* OR Favipiravir or T-705 or Avigan or FabiFlu or Pimodivir or VX-787 or JNJ-63623872 or AL-794 or ALS-033719 or ZSP1273 or Enisamium iodide or FAV00A or TG-1000 or GP681) OR AB (Viral Polymerase Complex Inhibitor* OR Favipiravir or T-705 or Avigan or FabiFlu or Pimodivir or VX-787 or JNJ-63623872 or AL-794 or ALS-033719 or ZSP1273 or Enisamium iodide or FAV00A or TG-1000 or GP681)
S31	TI (oseltamivir or tamiflu or "GS 4104" or GS4104 or GS-4104 or "GS 4071" or GS4071 or GS-4071 or zanamivir or relenza or "GG 167" or GG167 or GG-167 or CS-8958 or Dectova or Laninamivir or R-125489 or R125489 or "R 125489" or Inavir or peramivir or "BCX 1812" or BCX1812 or BCX-1812 or "RWJ 270201" or RWJ270201 or RWJ-270201 or Rapivab or rapiacta) OR AB (oseltamivir or tamiflu or "GS 4104" or GS4104 or GS-4104 or "GS 4071" or GS4071 or GS-4071 or zanamivir or relenza or "GG 167" or GG167 or GG-167 or CS-8958 or Dectova or Laninamivir or R-125489 or R125489 or "R 125489" or Inavir or peramivir or "BCX 1812" or BCX1812 or BCX-1812 or "RWJ 270201" or RWJ270201 or RWJ-270201 or Rapivab or rapiacta)
S30	(MH "Oseltamivir")
S29	TI(neuraminidase inhibitor* or NA inhibitor*) OR AB(neuraminidase inhibitor* or NA inhibitor*)
S28	TI Antiviral* OR AB Antiviral*
S27	(MH "Antiviral Agents")
S26	S24 OR S25
S25	TI (Influenza or flu or H1N1 or PH1N1 or H3N2 or AH1N1 or AH3N2 or H5N1 or H7N9) OR AB (Influenza or flu or H1N1 or PH1N1 or H3N2 or AH1N1 or AH3N2 or H5N1 or H7N9)
S24	(MH "Influenza+") OR (MH "Influenza A Virus+") OR (MH "Influenzavirus C") OR (MH "Influenza B Virus")
S23	S22 NOT S21
S22	S1 OR S2 OR S3 OR S4 OR S5 OR S6 OR S7 OR S8 OR S9 OR S10 OR S11 OR S12 OR S13 OR S14 OR S15
S21	S19 NOT S20
S20	MH (human)
S19	S16 OR S17 OR S18
S18	TI (animal model*)

S17	MH (animal studies)
S16	MH animals+
S15	AB (cluster W3 RCT)
S14	MH (crossover design) OR MH (comparative studies)
S13	AB (control W5 group)
S12	PT (randomized controlled trial)
S11	MH (placebos)
S10	MH (sample size) AND AB (assigned OR allocated OR control)
S9	TI (trial)
S8	AB (random*)
S7	TI (randomised OR randomized)
S6	MH cluster sample
S5	MH pretest-posttest design
S4	MH random assignment
S3	MH single-blind studies
S2	MH double-blind studies
S1	MH randomized controlled trials

Epistemonikos

Influenza antivirals

ClinIcaltrial.gov
Influenza antivirals

Appendix 2. Details of methods

2.1. Details of data extraction

Pairs of reviewers independently extracted the following data: study characteristics (first author, trial registration, publication year, publication status, country, and sample size); participant characteristics (age, sex, disease severity, comorbidities, influenza virus type); characteristics of antivirals (dosing, frequency, route of administration, treatment duration, and length of follow-up); and outcomes. Reviewers checked for duplicate data and resolved discrepancies by discussion or, if necessary, through consultation with a third reviewer.

2.2. Details of risk of bias assessment

To evaluate the risk of bias of eligible RCTs, we used a modified Cochrane risk of bias tool, including assessing the following domains: random sequence generation; allocation concealment; blinding of participants, healthcare providers, data collectors, outcome assessor/adjudicator, and data analysts; incomplete outcome data (≥ 10% missing data was considered high risk of bias); selective outcome reporting; and other sources of bias (i.e. baseline imbalance, early trial discontinuation). Pairs of reviewers independently rated each domain at the outcome level as: high, probably high, probably low, or low risk of bias. Because lack of blinding is unlikely to bias assessment of mortality, admission to ICU, progression to invasive mechanical ventilation, and emergence of antiviral resistance, we rated the blinding for these outcomes as low risk of bias, regardless of blinding status. Reviewers resolved discrepancies by discussion or, if necessary, with adjudication by a third party.

Appendix 3. Additional characteristics of eligible RCTs

Study	Comorbidities %	Pregnant %	Inpatient %	Intensive care %	Patients received influenza vaccination %	Details of standard care
Chen 2020	NR	NR	100	NR	0	NA
Dawood 2016	16.67 (asthma)	0	100	3.33	NR	NA
de Jong 2014	18.18 (COPD or other chronic lung disease), 4.96 (history of congestive heart failure or angina), 8.26 (diabetes)	0	100	19.01	4.96	Institutional standard care without neuraminidase inhibitor
Ison 2003	41.46 (pulmonary disease), 60.98 (heart disease), 19.51 (diabetes)	0	100	14.63	NR	NA
Ison 2013	19.67 (COPD/chronic lung disease), 7.28 (cardiac disease), 13.93 (diabetes)	0	100	NR	NR	NA
Kumar 2022	NR	0	100	13.55	NR	NA
Marty 2017	21.14 (COPD), 14.63 (asthma), 10.73 (coronary artery disease), 8.46 (arrhythmia), 24.88 (diabetes), 45.69 (hypertension)	0	100	39.67	10.89	NA
Ramirez 2018	NR	0	100	NR	NR	The standard care was provided according to the clinical management of the primary physician. This included the early administration of empiric antibiotic therapy and other supportive measures as deemed necessary by the attending physician.

NA, not applicable; NR, not reported.

Appendix 4. Risk of bias for eligible studies

Study	Sequence generation	Allocation concealment	Blinding of patients	Blinding of health care providers	Blinding of data collectors	Blinding of outcome assessors/ adjudicators	Blinding of data analysts	Incomplete outcome data	Selective outcome reporting	Other bias
**Mortality**										
de Jong 2014	Probably Low	Probably High	Low	Low	Low	Low	Low	Low	Probably Low	Low
Ison 2003	Low	Low	Low	Low	Low	Low	Low	High	Probably Low	Probably High
Ison 2013	Probably Low	Low	Low	Low	Low	Low	Low	Low	Low	Low
Kumar 2022	Low	Low	Low	Low	Low	Low	Low	Low	Low	Low
Marty 2017	Low	Low	Low	Low	Low	Low	Low	High	Low	Low
Ramirez 2018	Low	Probably Low	Low	Low	Low	Low	Low	Low	Low	Low
**Admission to ICU**										
de Jong 2014	Probably Low	Probably High	Low	Low	Low	Low	Low	Low	Low	Low
Ison 2013	Probably Low	Low	Low	Low	Low	Low	Low	Low	Low	Low
Kumar 2022	Low	Low	Low	Low	Low	Low	Low	Low	Low	Low
**Progression to mechanical ventilation**										
Kumar 2022	Low	Low	Low	Low	Low	Low	Low	Low	Probably Low	Low
Marty 2017	Low	Low	Low	Low	Low	Low	Low	High	Probably Low	Low
**Emergence of resistance**										
Kumar 2022	Low	Low	Low	Low	Low	Low	Low	Low	Low	Low

Marty 2017	Low	Low	Low	Low	Low	Low	Low	High	Low	Low
**Any adverse events**										
Ison 2003	Low	Low	Low	Low	Probably High	Probably High	Probably High	High	Probably Low	Probably High
Ison 2013	Probably Low	Low	Low	Low	Probably High	Probably High	Low	Low	Low	Low
Kumar 2022	Low	Low	Low	Low	Low	Low	Low	Low	Low	Low
Marty 2017	Low	Low	Low	Low	Probably High	Low	Probably High	High	Low	Low
**Adverse events related to treatments**										
Kumar 2022	Low	Low	Low	Low	Low	Low	Low	Low	Low	Low
Marty 2017	Low	Low	Low	Low	Probably High	Low	Probably High	High	Low	Low
**Serious adverse events**										
Ison 2003	Low	Low	Low	Low	Probably High	Probably High	Probably High	High	Probably Low	Probably High
Ison 2013	Probably Low	Low	Low	Low	Probably High	Probably High	Low	Low	Low	Low
Kumar 2022	Low	Low	Low	Low	Low	Low	Low	Low	Low	Low
Marty 2017	Low	Low	Low	Low	Probably High	Low	Probably High	High	Low	Low
**Duration of hospitalization**										
Dawood 2016	Low	Low	Low	Low	Probably High	Probably High	Probably High	Low	Low	Probably High
Ison 2003	Low	Low	Low	Low	Probably High	Probably High	Probably High	High	Probably Low	Probably High
Ison 2013	Probably Low	Low	Low	Low	Probably High	Probably High	Low	Low	Low	Low
Kumar 2022	Low	Low	Low	Low	Low	Low	Low	Low	Low	Low
Ramirez 2018	Low	Probably Low	High	High	High	High	High	Low	Low	Low

**Time to alleviation of symptoms**										
Chen 2020	Low	Probably High	Probably High	Probably High	Probably High	Probably High	Probably High	Low	Low	Low
de Jong 2014	Probably Low	Probably High	Low	Low	Probably Low	Probably Low	Probably High	Low	Low	Low
Ison 2013	Probably Low	Low	Low	Low	Probably High	Probably High	Low	Low	Low	Low
**Duration of mechanical ventilation**										
Kumar 2022	Low	Low	Low	Low	Low	Low	Low	Low	Low	Low
Marty 2017	Low	Low	Low	Low	Probably High	Low	Low	High	Probably Low	Low

Appendix 5. Network plots

*The size of the circle represents the number of participants. The width of the line represents the number of studies.

5.1. Network plot for admission to ICU

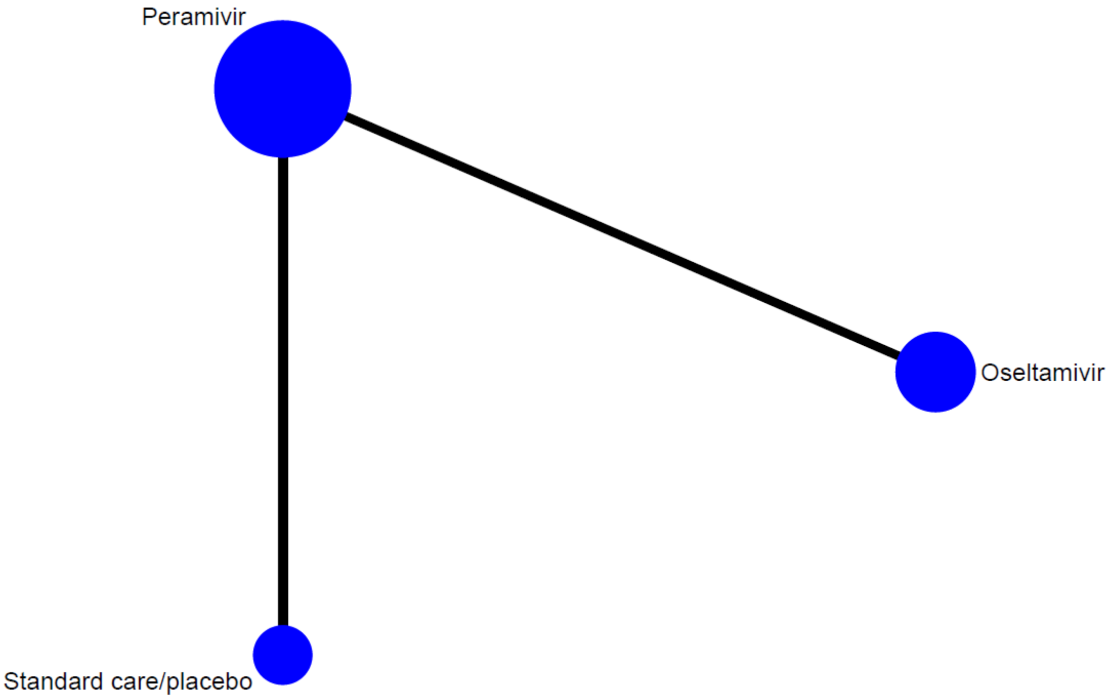

5.2. Network plot for duration of hospitalization

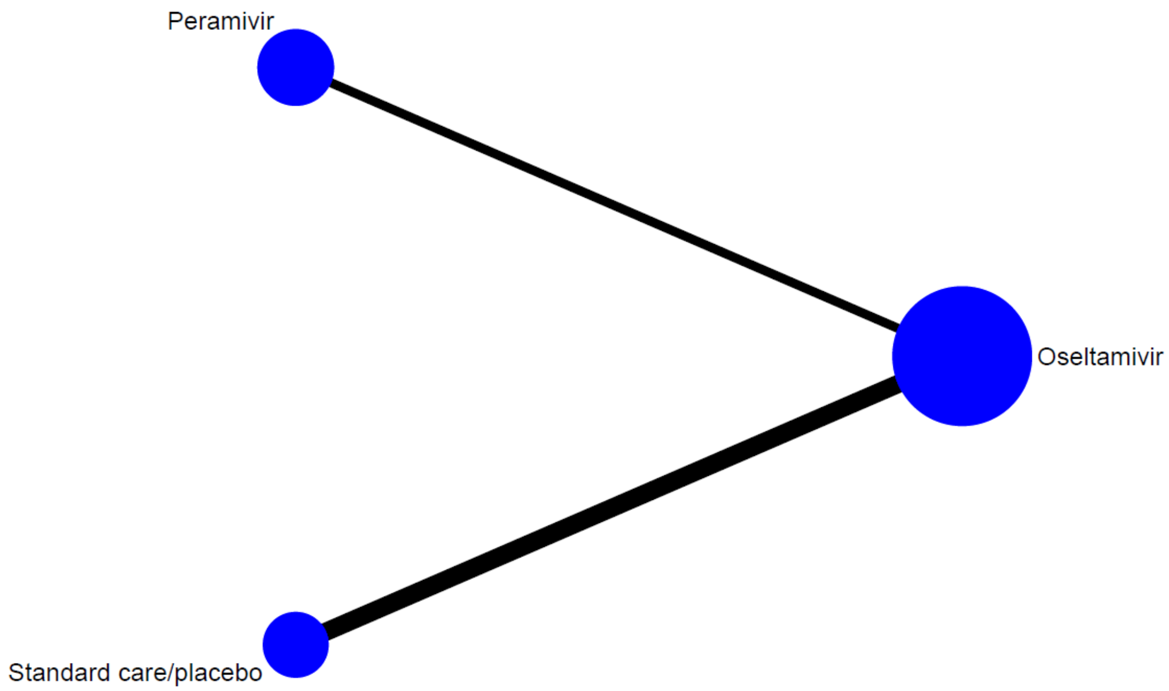

5.3. Network plot for time to alleviation of symptoms

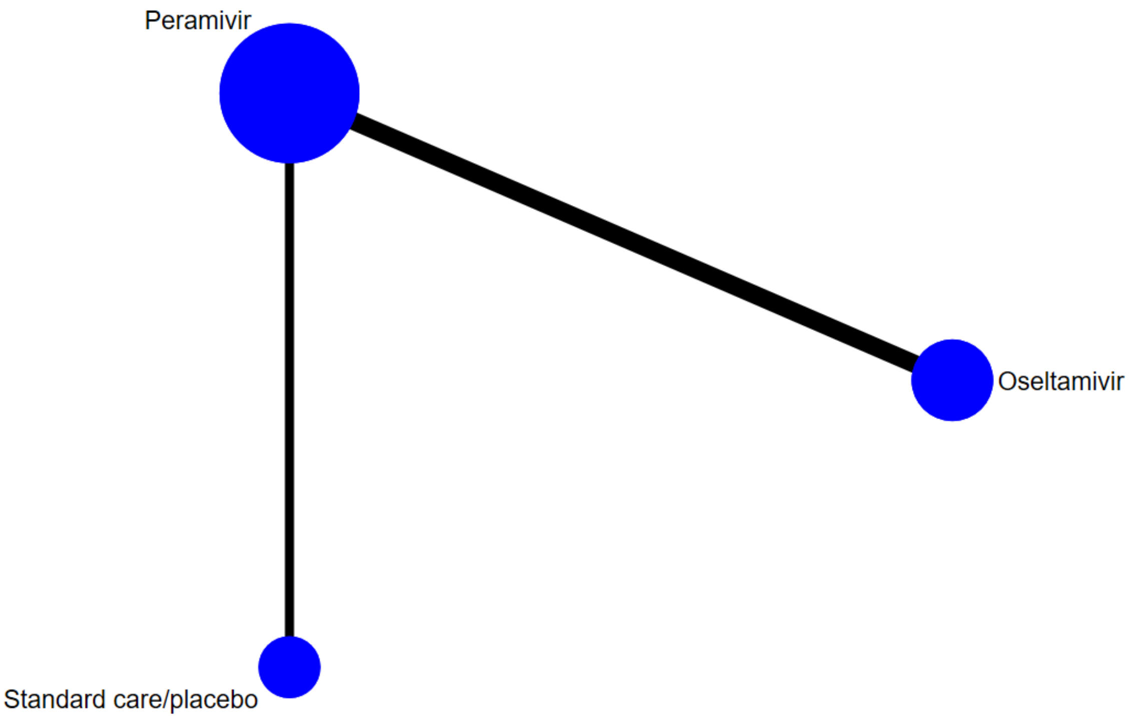

5.4. Network plot for any adverse events

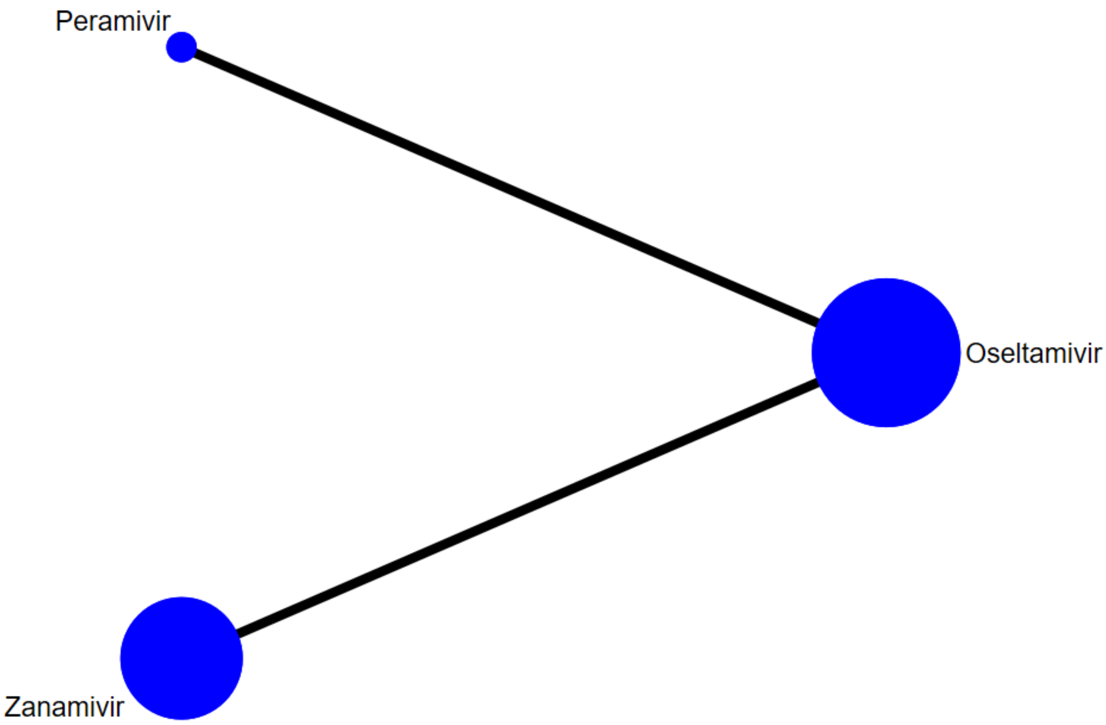

5.5. Network plot for serious adverse events

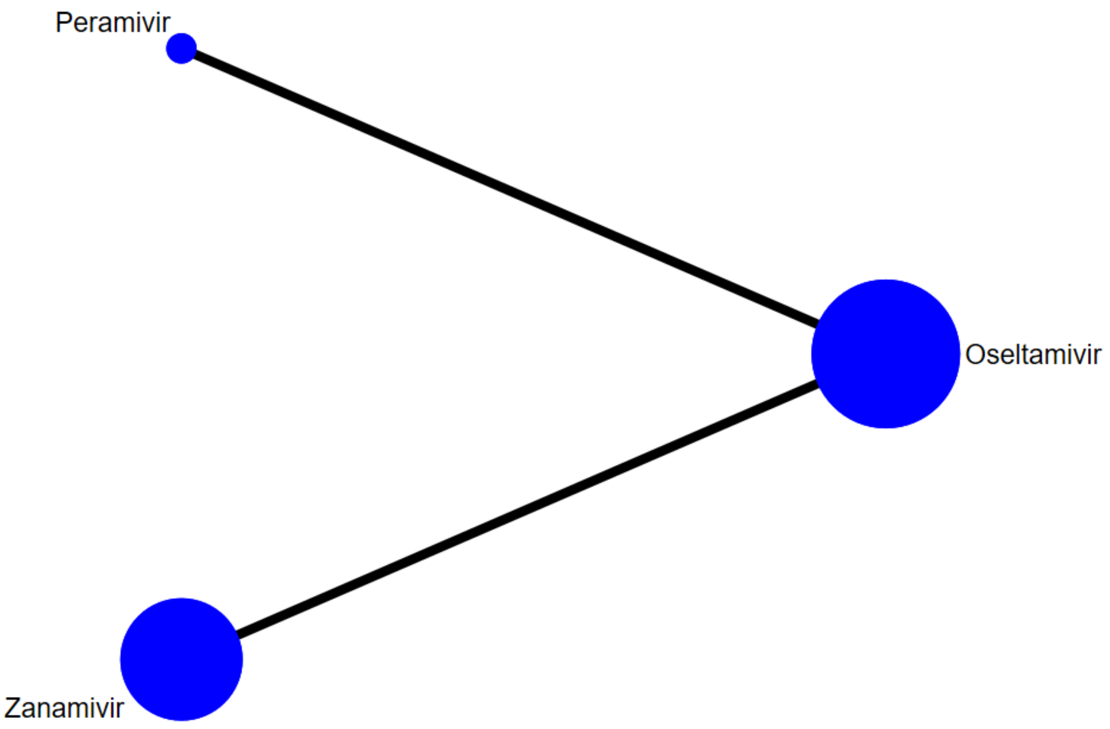

Appendix 6. Assessment of between-study heterogeneity

Outcome	Comparison	No. study	I^2
Mortality	Oseltamivir vs. Peramivir	1	NA
	Oseltamivir vs. Standard care/placebo	1	NA
	Oseltamivir vs. Zanamivir	1	NA
	Peramivir vs. Standard care/placebo	1	NA
Admission to ICU	Oseltamivir vs. Peramivir	1	NA
	Peramivir vs. Standard care/placebo	1	NA
Time to alleviation of symptoms	Oseltamivir vs. Peramivir	2	0%
	Peramivir vs. Standard care/placebo	1	NA
Duration of hospitalization	Oseltamivir vs. Standard care/placebo	2	0%
	Oseltamivir vs. Peramivir	1	NA
Any adverse events	Oseltamivir vs. Peramivir	1	NA
	Oseltamivir vs. Zanamivir	1	NA
Serious adverse events	Oseltamivir vs. Peramivir	1	NA
	Oseltamivir vs. Zanamivir	1	NA

NA, not applicable.

Appendix 7. Assessment of global incoherence

Outcome	P value
Mortality	0.487
Admission to ICU	NA
Time to alleviation of symptoms	NA
Duration of hospitalization	NA
Any adverse events	NA
Serious adverse events	NA

NA, not applicable.

Appendix 8. Direct, indirect, and network treatment estimates

8.1. Direct, indirect, and network treatment estimates for mortality

Comparison	k	Prop	NMA (95% CI)	Direct (95% CI)	Indirect (95% CI)	RoR (95% CI)	z	Incoherence p-value
Oseltamivir vs. Peramivir	1	0.61	1.33 (0.11 to 15.87)	0.66 (0.03 to 15.79)	3.97 (0.08 to 207.32)	0.17 (0.00 to 26.45)	-0.7	0.487
Oseltamivir vs. Standard care/placebo	1	0.78	0.53 (0.07 to 4.24)	0.78 (0.07 to 8.17)	0.13 (0.00 to 11.50)	6.05 (0.04 to 969.21)	0.7	0.487
Oseltamivir vs. Zanamivir	1	1	0.91 (0.44 to 1.87)	0.91 (0.44 to 1.87)	NA	NA	NA	NA
Peramivir vs. Standard care/placebo	1	0.61	0.40 (0.03 to 4.72)	0.20 (0.01 to 4.69)	1.18 (0.02 to 61.91)	0.17 (0.00 to 26.45)	-0.7	0.487
Peramivir vs. Zanamivir	0	0	0.68 (0.05 to 9.01)	NA	0.68 (0.05 to 9.01)	NA	NA	NA
Zanamivir vs. Standard care/placebo	0	0	0.58 (0.06 to 5.29)	NA	0.58 (0.06 to 5.29)	NA	NA	NA

Comparison: treatment comparison; k: number of studies providing direct evidence; prop: direct evidence proportion; NMA: estimated treatment effect (RR) in network meta-analysis; direct: estimated treatment effect (RR) derived from direct evidence; indirect: estimated treatment effect (RR) derived from indirect evidence; RoR: Ratio of Ratios (direct versus indirect); z: z-value of test for disagreement (direct versus indirect); Incoherence p-value: p-value of test for disagreement (direct versus indirect). NA: not applicable.

8.2. Direct, indirect, and network treatment estimates for admission to ICU

Comparison	k	Prop	NMA (95% CI)	Direct (95% CI)	Indirect (95% CI)	Diff (95% CI)	z	Incoherence p-value
Oseltamivir vs. Peramivir	1	1	0.043 (-0.034 to 0.121)	0.043 (-0.034 to 0.121)	NA	NA	NA	NA
Oseltamivir vs. Standard care/placebo	0	0	0.015 (-0.089 to 0.118)	NA	0.015 (-0.089 to 0.118)	NA	NA	NA
Peramivir vs. Standard care/placebo	1	1	-0.029 (-0.097 to 0.040)	-0.029 (-0.097 to 0.040)	NA	NA	NA	NA

Comparison: treatment comparison; k: number of studies providing direct evidence; prop: direct evidence proportion; NMA: estimated treatment effect (RD) in network meta-analysis; direct: estimated treatment effect (RD) derived from direct evidence; indirect: estimated treatment effect (RD) derived from indirect evidence; Diff: difference between direct and indirect treatment estimates; z: z-value of test for disagreement (direct versus indirect); Incoherence p-value: p-value of test for disagreement (direct versus indirect). NA: not applicable.

8.3. Direct, indirect, and network treatment estimates for duration of hospitalization

Comparison	k	Prop	NMA (95% CI)	Direct (95% CI)	Indirect (95% CI)	Diff (95% CI)	z	Incoherence p-value
Oseltamivir vs. Peramivir	1	1	0.1 (-0.98 to 1.18)	0.10 (-0.98 to 1.18)	NA	NA	NA	NA
Oseltamivir vs. Standard care/placebo	2	1	-1.63 (-2.81 to -0.45)	-1.63 (-2.81 to -0.45)	NA	NA	NA	NA
Peramivir vs. Standard care/placebo	0	0	-1.73 (-3.33 to -0.13)	NA	-1.73 (-3.33 to -0.13)	NA	NA	NA

Comparison: treatment comparison; k: number of studies providing direct evidence; prop: direct evidence proportion; NMA: estimated treatment effect (MD) in network meta-analysis; direct: estimated treatment effect (MD) derived from direct evidence; indirect: estimated treatment effect (MD) derived from indirect evidence; Diff: difference between direct and indirect treatment estimates; z: z-value of test for disagreement (direct versus indirect); Incoherence p-value: p-value of test for disagreement (direct versus indirect). NA: not applicable.

8.4. Direct, indirect, and network treatment estimates for time to alleviation of symptoms

Comparison	k	Prop	NMA (95% CI)	Direct (95% CI)	Indirect (95% CI)	Diff (95% CI)	z	Incoherence p-value
Oseltamivir vs. Peramivir	2	1	0.39 (-0.63 to 1.40)	0.39 (-0.63 to 1.40)	NA	NA	NA	NA
Oseltamivir vs. Standard care/placebo	0	0	0.34 (-0.86 to 1.54)	NA	0.34 (-0.86 to 1.54)	NA	NA	NA
Peramivir vs. Standard care/placebo	1	1	-0.05 (-0.69 to 0.59)	-0.05 (-0.69 to 0.59)	NA	NA	NA	NA

Comparison: treatment comparison; k: number of studies providing direct evidence; prop: direct evidence proportion; NMA: estimated treatment effect (MD) in network meta-analysis; direct: estimated treatment effect (MD) derived from direct evidence; indirect: estimated treatment effect (MD) derived from indirect evidence; Diff: difference between direct and indirect treatment estimates; z: z-value of test for disagreement (direct versus indirect); Incoherence p-value: p-value of test for disagreement (direct versus indirect). NA: not applicable.

8.5. Direct, indirect, and network treatment estimates for any adverse events

Comparison	k	Prop	NMA (95% CI)	Direct (95% CI)	Indirect (95% CI)	RoR (95% CI)	z	Incoherence p-value
Oseltamivir vs. Peramivir	1	1	0.77 (0.52 to 1.14)	0.77 (0.52 to 1.14)	NA	NA	NA	NA
Oseltamivir vs. Zanamivir	1	1	1.12 (0.99 to 1.28)	1.12 (0.99 to 1.28)	NA	NA	NA	NA
Peramivir vs. Zanamivir	0	0	1.46 (0.97 to 2.21)	NA	1.46 (0.97 to 2.21)	NA	NA	NA

Comparison: treatment comparison; k: number of studies providing direct evidence; prop: direct evidence proportion; NMA: estimated treatment effect (RR) in network meta-analysis; direct: estimated treatment effect (RR) derived from direct evidence; indirect: estimated treatment effect (RR) derived from indirect evidence; RoR: Ratio of Ratios (direct versus indirect); z: z-value of test for disagreement (direct versus indirect); Incoherence p-value: p-value of test for disagreement (direct versus indirect). NA: not applicable.

8.6. Direct, indirect, and network treatment estimates for serious adverse events

Comparison	k	Prop	NMA (95% CI)	Direct (95% CI)	Indirect (95% CI)	RoR (95% CI)	z	Incoherence p-value
Oseltamivir vs. Peramivir	1	1	0.79 (0.26 to 2.39)	0.79 (0.26 to 2.39)	NA	NA	NA	NA
Oseltamivir vs. Zanamivir	1	1	1.07 (0.75 to 1.53)	1.07 (0.75 to 1.53)	NA	NA	NA	NA
Peramivir vs. Zanamivir	0	0	1.35 (0.42 to 4.32)	NA	1.35 (0.42 to 4.32)	NA	NA	NA

Comparison: treatment comparison; k: number of studies providing direct evidence; prop: direct evidence proportion; NMA: estimated treatment effect (RR) in network meta-analysis; direct: estimated treatment effect (RR) derived from direct evidence; indirect: estimated treatment effect (RR) derived from indirect evidence; RoR: Ratio of Ratios (direct versus indirect); z: z-value of test for disagreement (direct versus indirect); Incoherence p-value: p-value of test for disagreement (direct versus indirect). NA: not applicable.

Appendix 9. GRADE summary of findings for outcomes

9.1. GRADE summary of findings for admission to ICU for different comparisons

Comparison	Study results and measurements	Absolute difference (95% CI)	Certainty in effect estimates	Plain language summary
Oseltamivir versus Standard care/placebo	Risk difference: 0.015 (95% CI -0.089 to 0.118) Based on indirect evidence	15 more per 1000 (95% CI 89 fewer to 118 more)	Very low†*	Whether oseltamivir reduces admission to ICU is very uncertain.
Peramivir versus Standard care/placebo	Risk difference: -0.029 (95% CI -0.097 to 0.040) Based on data from 98 participants in 1 study	29 fewer per 1000 (95% CI 97 fewer to 40 more)	Very low†‡	Whether peramivir reduces admission to ICU is very uncertain.
Oseltamivir versus Peramivir	Risk difference: 0.043 (95% CI -0.034 to 0.121) Based on data from 137 participants in 1 study	43 more per 1000 (95% CI 34 fewer to 121 more)	Very low*	Whether oseltamivir reduces admission to ICU compared with peramivir is very uncertain.

*Rated down 3 levels for imprecision.

†Rated down 1 level for risk of bias.

‡Rated down 2 levels for imprecision.

9.2. GRADE summary of findings for time to alleviation of symptoms for different comparisons

Comparison	Mean difference (95% CI)	Certainty in effect estimates	Plain language summary
Oseltamivir versus Standard care/placebo	0.34 (-0.86 to 1.54)	Low†‡	Oseltamivir may have little or no effect on time to alleviation of symptoms.
Peramivir versus Standard care/placebo	-0.05 (-0.69 to 0.59)	Low†‡	Peramivir may have little or no effect on time to alleviation of symptoms.
Oseltamivir versus Peramivir	0.39 (-0.63 to 1.40)	Low†‡	There may be little or no difference between oseltamivir and peramivir in time to alleviation of symptoms.

†Rated down 1 level for risk of bias.

‡Rated down 1 level for imprecision.

9.3. GRADE summary of findings for any adverse events for different comparisons

Comparison	Study results and measurements	Absolute effect estimates (per 1000)		Absolute difference (95% CI)	Certainty in effect estimates	Plain language summary
Oseltamivir versus Peramivir	Risk ratio: 0.77 (95% CI 0.52 to 1.14) Based on data from 137 participants in 1 study	Peramivir: 851	Oseltamivir: 655	196 fewer per 1000 (95% CI 408 fewer to 119 more)	Very low†‡	Whether oseltamivir increases any adverse events compared with peramivir is very uncertain.
Oseltamivir versus Zanamivir	Risk ratio: 1.12 (95% CI 0.99 to 1.28) Based on data from 615 participants in 1 study	Zanamivir: 583	Oseltamivir: 653	70 more per 1000 (95% CI 6 fewer to 163 more)	Very low†‡	Whether oseltamivir increases any adverse events compared with zanamivir is very uncertain.
Peramivir versus Zanamivir	Risk ratio: 1.46 (95% CI 0.97 to 2.21) Based on indirect evidence	Zanamivir: 583	Peramivir: 851	268 more per 1000 (95% CI 17 fewer to 417 more)	Very low†‡	Whether peramivir increases any adverse events compared with zanamivir is very uncertain.

†Rated down 1 level for risk of bias.

‡Rated down 2 levels for imprecision.

9.4. GRADE summary of findings for serious adverse events for different comparisons

Comparison	Study results and measurements	Absolute effect estimates (per 1000)		Absolute difference (95% CI)	Certainty in effect estimates	Plain language summary
Oseltamivir versus Peramivir	Risk ratio: 0.79 (95% CI 0.26 to 2.39) Based on data from 137 participants in 1 study	Peramivir: 234	Oseltamivir: 185	49 fewer per 1000 (95% CI 173 fewer to 325 more)	Very low†‡	Whether oseltamivir increases serious adverse events compared with peramivir is very uncertain.
Oseltamivir versus Zanamivir	Risk ratio: 1.07 (95% CI 0.75 to 1.53) Based on data from 615 participants in 1 study	Zanamivir: 173	Oseltamivir: 185	12 more per 1000 (95% CI 43 fewer to 92 more)	Very low†‡	Whether oseltamivir increases serious adverse events compared with zanamivir is very uncertain.
Peramivir versus Zanamivir	Risk ratio: 1.35 (95% CI 0.42 to 4.32) Based on indirect evidence	Zanamivir: 173	Peramivir: 234	61 more per 1000 (95% CI 100 fewer to 574 more)	Very low†‡	Whether peramivir increases serious adverse events compared with zanamivir is very uncertain.

†Rated down 1 level for risk of bias.

‡Rated down 2 levels for imprecision.

9.5. GRADE summary of findings for progression to mechanical ventilation, emergence of resistance, and adverse events related to treatments

Outcomes	Comparison	Study results and measurements	Absolute effect estimates (per 1000)		Absolute difference (95% CI)	Certainty in effect estimates	Plain language summary
Progression to mechanical ventilation	Oseltamivir versus Zanamivir	Risk ratio: 1.20 (95% CI 0.90 to 1.62) Based on data from 488 participants in 1 study	Zanamivir: 255	Oseltamivir: 306	51 more per 1000 (95% CI 26 fewer to 158 more)	Very low†‡	Whether oseltamivir reduces progression to mechanical ventilation compared with zanamivir is very uncertain.
Emergence of resistance	Oseltamivir versus Zanamivir	Risk ratio: 2.89 (95% CI 0.88 to 9.49) Based on data from 615 participants in 1 study	Zanamivir: 10	Oseltamivir: 29	19 more per 1000 (95% CI 1 fewer to 85 more)	Very low†‡	Whether oseltamivir increases emergence of resistance compared with zanamivir is very uncertain.
Adverse events related to treatments	Oseltamivir versus Zanamivir	Risk ratio: 1.49 (95% CI 1.00 to 2.23) Based on data from 615 participants in 1 study	Zanamivir: 115	Oseltamivir: 171	56 more per 1000 (95% CI 0 fewer to 141 more)	Very low†‡	Whether oseltamivir increases adverse events related to treatments compared with zanamivir is very uncertain.

†Rated down 1 level for risk of bias.

‡Rated down 2 levels for imprecision.

9.6. GRADE summary of findings for duration of mechanical ventilation

Comparison	Mean difference (95% CI)	Certainty in effect estimates	Plain language summary
Oseltamivir versus Zanamivir	0.89 (-2.32 to 4.10)	Very low†‡	Whether oseltamivir reduces duration of mechanical ventilation compared with zanamivir is very uncertain.

†Rated down 1 level for risk of bias.

‡Rated down 2 levels for imprecision.

Appendix 10. Results of a study, comparing baloxavir plus NAIs with NAIs, not included in the network meta-analysis

10.1. Forest plots for baloxavir plus NAIs versus NAIs (oseltamivir, zanamivir, or peramivir)

Dichotomous outcomes

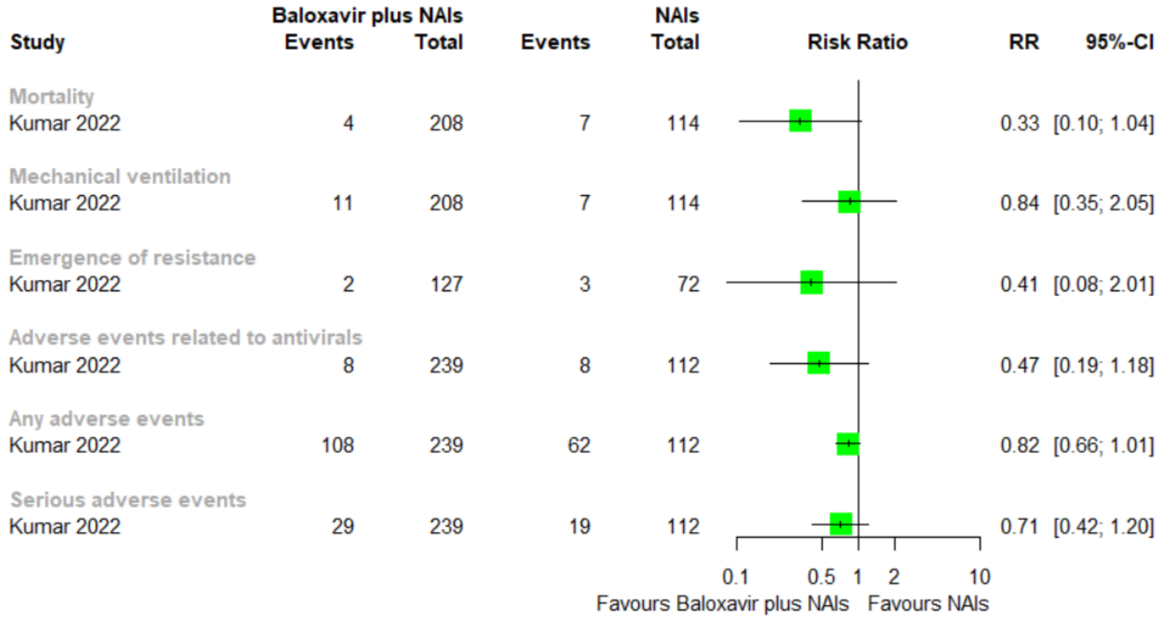

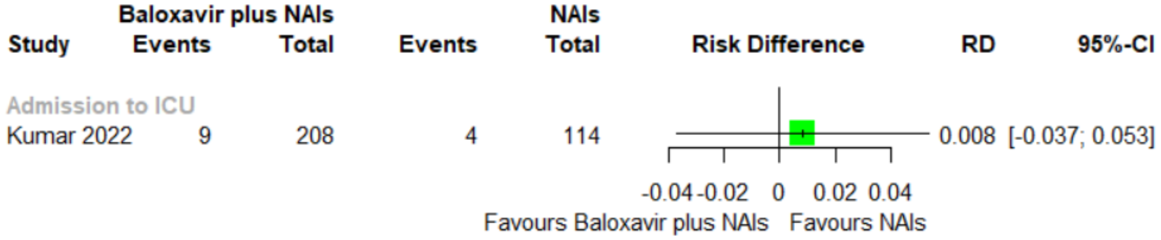

Continuous outcomes

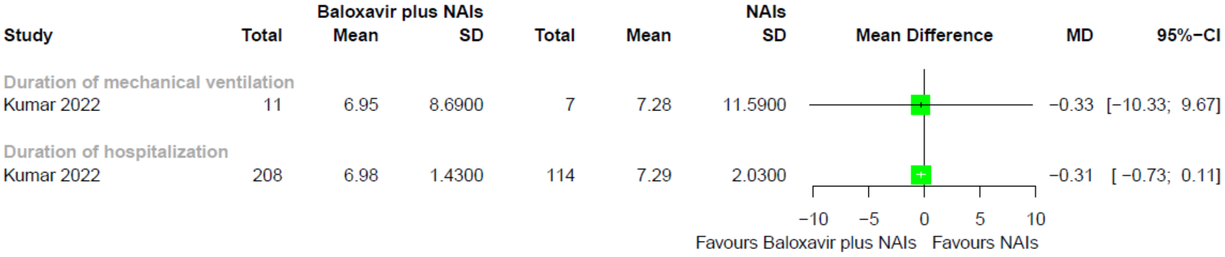

10.2. GRADE summary of findings for baloxavir plus NAIs versus NAIs (oseltamivir, zanamivir, or peramivir)

**Outcome** Timeframe	**Study results and measurements**	**Absolute effect estimates** NAIs	**Absolute effect estimates** Baloxavir plus NAIs	**Certainty of the Evidence** (Quality of evidence)	**Summary**
Mortality (Seasonal influenza)	Risk ratio: 0.33 (95% CI 0.10 - 1.04) Based on data from 322 participants in 1 study	**15** per 1000	**5** per 1000	**Very low** Due to extremely serious imprecision[1]	Whether baloxavir plus NAIs reduces mortality in people with seasonal influenza compared with NAIs is very uncertain.
		Difference: **10 fewer per 1000** (95% CI 13 fewer - 1 more)			
Mortality (Zoonotic influenza)	Risk ratio: 0.33 (95% CI 0.1 - 1.04) Based on data from 322 participants in 1 study	**195** per 1000	**64** per 1000	**Very low** Due to extremely serious imprecision[1]	Whether baloxavir plus NAIs reduces mortality in people with zoonotic influenza compared with NAIs is very uncertain.
		Difference: **131 fewer per 1000** (95% CI 175 fewer - 8 more)			
Admission to ICU	Risk difference: 0.008 (95% CI -0.037 - 0.053) Based on data from 322 participants in 1 study	**35** per 1000	**43** per 1000	**Very low** Due to extremely serious imprecision[1]	Whether baloxavir plus NAIs reduces admission to ICU compared with NAIs is very uncertain.
		Difference: **8 more per 1000** (95% CI 37 fewer - 53 more)			
Mechanical ventilation	Risk ratio: 0.84 (95% CI 0.35 - 2.05) Based on data from 322 participants in 1 study	**61** per 1000	**51** per 1000	**Very low** Due to extremely serious imprecision[1]	Whether baloxavir plus NAIs reduces mechanical ventilation compared with NAIs is very uncertain.
		Difference: **10 fewer per 1000** (95% CI 40 fewer - 64 more)			
Any adverse events	Risk ratio: 0.82 (95% CI 0.66 - 1.01) Based on data from 351 participants in 1 study	**554** per 1000	**454** per 1000	**Very low** Due to extremely serious imprecision[1]	Whether baloxavir plus NAIs increases any adverse events compared with NAIs is very uncertain.
		Difference: **100 fewer per 1000** (95% CI 188 fewer - 6 more)			
Adverse events related to treatment	Risk ratio: 0.47 (95% CI 0.19 - 1.18) Based on data from 351 participants in 1 study	**71** per 1000	**33** per 1000	**Very low** Due to extremely serious imprecision[1]	Whether baloxavir plus NAIs increases adverse events related to treatment compared with NAIs is very uncertain.
		Difference: **38 fewer per 1000** (95% CI 58 fewer - 13 more)			
Serious adverse events	Risk ratio: 0.71 (95% CI 0.42 - 1.2) Based on data from 351 participants in 1 study	**170** per 1000	**121** per 1000	**Very low** Due to extremely serious imprecision[1]	Whether baloxavir plus NAIs increases serious adverse events compared with NAIs is very uncertain.
		Difference: **49 fewer per 1000** (95% CI 99 fewer - 34 more)			
Emergence of resistance	Risk ratio: 0.41 (95% CI 0.08 - 2.01) Based on data from 199	**42** per 1000	**17** per 1000	**Low** Due to very serious imprecision[2]	Baloxavir plus NAIs may have little or no effect on emergence of resistance

	participants in 1 study	Difference: **25 fewer per 1000** (95% CI 39 fewer - 42 more)			compared with NAIs.
Duration of hospitalization	Measured by: day<br>Lower better<br>Based on data from 322 participants in 1 study	**7.29** Mean	**6.98** Mean	**Low**<br>Due to very serious imprecision[2]	Baloxavir plus NAIs may have little or no effect on duration of hospitalization compared with NAIs.
		Difference: **MD 0.31 lower** (95% CI 0.73 lower - 0.11 higher)			
Duration of mechanical ventilation	Measured by: day<br>Lower better<br>Based on data from 18 participants in 1 study	**7.28** Mean	**6.95** Mean	**Very low**<br>Due to extremely serious imprecision[1]	Whether baloxavir plus NAIs reduces duration of mechanical ventilation compared with NAIs is very uncertain.
		Difference: **MD 0.33 lower** (95% CI 10.33 lower - 9.67 higher)			

1. **Imprecision: extremely serious.** Wide confidence intervals, only data from one study
2. **Imprecision: very serious.** Only data from one study

Appendix 11. Results of a study, comparing zanamivir plus rimantadine with rimantadine, not included in the network meta-analysis

11.1. Forest plots for zanamivir plus rimantadine versus rimantadine

Dichotomous outcomes

Study	Zanamivir plus rimantadine Events	Zanamivir plus rimantadine Total	Rimantadine Events	Rimantadine Total	Risk Ratio	RR	95%−CI
Mortality							
Ison 2003	2	20	1	21		1.75	[0.25; 12.06]
Any adverse events							
Ison 2003	14	20	16	21		0.92	[0.64; 1.32]
Serious adverse events							
Ison 2003	3	20	4	21		0.82	[0.23; 2.87]

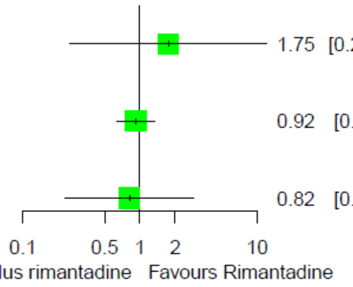

Continuous outcomes- Duration of hospitalization

Study	Zanamivir plus rimantadine Total	Zanamivir plus rimantadine Mean	Zanamivir plus rimantadine SD	Rimantadine Total	Rimantadine Mean	Rimantadine SD	Mean Difference	MD	95%−CI
Ison 2003	16	4.70	2.3000	20	5.20	2.3000		−0.50	[−2.01; 1.01]

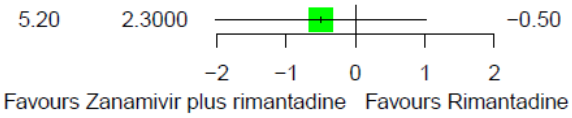

11.2. GRADE summary of findings for zanamivir plus rimantadine versus rimantadine

**Outcome** Timeframe	**Study results and measurements**	**Absolute effect estimates**		**Certainty of the Evidence** (Quality of evidence)	**Summary**
		Rimantadine	Zanamivir plus rimantadine		
Mortality (Seasonal influenza)	Risk ratio: 1.75 (95% CI 0.25 - 12.06) Based on data from 41 participants in 1 study	**24** per 1000	**42** per 1000	**Very low** Due to serious risk of bias, Due to extremely serious imprecision[1]	Whether zanamivir plus rimantadine reduces mortality in people with seasonal influenza compared with rimantadine is very uncertain.
		Difference: **18 more per 1000** (95% CI 18 fewer - 265 more)			
Mortality (Seasonal influenza)	Risk ratio: 1.75 (95% CI 0.25 - 12.06) Based on data from 41 participants in 1 study	**310** per 1000	**543** per 1000	**Very low** Due to serious risk of bias, Due to extremely serious imprecision[1]	Whether zanamivir plus rimantadine reduces mortality in people with zoonotic influenza compared with rimantadine is very uncertain.
		Difference: **233 more per 1000** (95% CI 232 fewer - 690 more)			
Any adverse events	Risk ratio: 0.92 (95% CI 0.64 - 1.32) Based on data from 41 participants in 1 study	**762** per 1000	**701** per 1000	**Very low** Due to serious risk of bias, Due to extremely serious imprecision[1]	Whether zanamivir plus rimantadine increases any adverse events compared with rimantadine is very uncertain.
		Difference: **61 fewer per 1000** (95% CI 274 fewer - 244 more)			
Serious adverse events	Risk ratio: 0.82 (95% CI 0.23 - 2.87) Based on data from 41 participants in 1 study	**190** per 1000	**156** per 1000	**Very low** Due to serious risk of bias, Due to extremely serious imprecision[1]	Whether zanamivir plus rimantadine increases any adverse events compared with rimantadine is very uncertain.
		Difference: **34 fewer per 1000** (95% CI 146 fewer - 355 more)			
Duration of hospitalization	Measured by: day Lower better Based on data from 36 participants in 1 study	**5.20** Mean	**4.70** Mean	**Very low** Due to serious risk of bias, Due to very serious imprecision[2]	Whether zanamivir plus rimantadine reduces duration of hospitalization compared with rimantadine is very uncertain.
		Difference: **MD 0.50 lower** (95% CI 2.01 lower - 1.01 higher)			

1. **Risk of Bias: serious.** Trials stopping earlier than scheduled, resulting in potential for overestimating benefits, Incomplete data and/or large loss to follow up; **Imprecision: extremely serious.** Very wide confidence intervals, only data from one study
2. **Risk of Bias: serious.** Trials stopping earlier than scheduled, resulting in potential for overestimating benefits, Incomplete data and/or large loss to follow up; **Imprecision: very serious.** Wide confidence intervals, only data from one study
